# Coralysis enables sensitive identification of imbalanced cell types and states in single-cell data via multi-level integration

**DOI:** 10.1093/nar/gkaf1128

**Published:** 2025-11-13

**Authors:** António G G Sousa, Johannes Smolander, Sini Junttila, Laura L Elo

**Affiliations:** Turku Bioscience Centre, University of Turku and Åbo Akademi University, Turku 20520, Finland; InFLAMES Research Flagship Centre, University of Turku, Turku 20520, Finland; Turku Bioscience Centre, University of Turku and Åbo Akademi University, Turku 20520, Finland; InFLAMES Research Flagship Centre, University of Turku, Turku 20520, Finland; Turku Bioscience Centre, University of Turku and Åbo Akademi University, Turku 20520, Finland; InFLAMES Research Flagship Centre, University of Turku, Turku 20520, Finland; Turku Bioscience Centre, University of Turku and Åbo Akademi University, Turku 20520, Finland; InFLAMES Research Flagship Centre, University of Turku, Turku 20520, Finland; Institute of Biomedicine, University of Turku, Turku 20520, Finland

## Abstract

Complex single-cell analyses now routinely integrate multiple datasets, followed by cell-type annotation and differential expression analysis. Current state-of-the-art integration methods often struggle with imbalanced cell types across datasets particularly when highly similar but distinct cell types are not present in all datasets. Inaccurate integration leads to incorrect annotations, affecting downstream analyses such as differential expression. To streamline single-cell data analysis, we introduce Coralysis, an all-in-one package featuring a sensitive integration algorithm, reference-mapping for accurate automatic annotation, and fine-grained cell-state identification. We demonstrate that Coralysis shows consistently high performance across diverse integration tasks, outperforming state-of-the-art methods particularly in challenging settings when similar cell types are imbalanced or missing. It accurately predicts cell-type identities across various annotation scenarios. A key strength of Coralysis is its ability to provide cell-specific probability scores, enabling the identification of transient and stable cell-states, along with their differential expression patterns. Importantly, Coralysis performs robustly on different types of single-cell data from transcriptomics to proteomics. Overall, Coralysis includes all the main steps of single-cell data analysis; it preserves subtle biological variation by improving the integration and annotation of imbalanced cell types, and identifies fine-grained cell-states—enabling a faithful analysis of the cellular landscape in complex single-cell experiments.

## Graphical Abstract

**Graphical Abstract:**
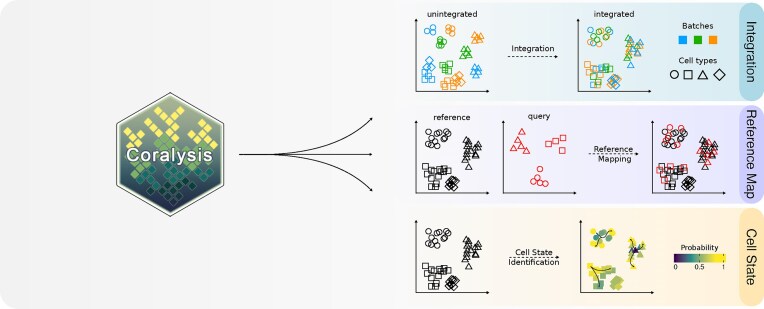

## Introduction

Single-cell transcriptomes from multiple subjects across different biological conditions can provide valuable insights into various biological phenomena, such as cell trajectories [1] in embryonic development and differences in gene expression [2] between healthy and diseased individuals. In analysing such datasets, a typical goal is to project and cluster the multi-subject single-cell transcriptomes into a low-dimensional space, ensuring that cells of the same type cluster together. However, technical noise introduced by differences in the procedures for handling sets of samples (i.e. in batches) can distort the biological signal, leading to cells being clustered based on their batch identity rather than their cell type. Correcting for such biases resulting from technical batch effects by integrating and “aligning” cells across batches is crucial to preserve the biological signal and accurately cluster the same cell types together.

A few benchmarks have been performed to evaluate horizontal integration methods for single-cell RNA sequencing (scRNA-seq) [3–5]. These benchmarks differ in the type of data used, the complexity of the integration tasks tested, and the performance metrics calculated. For example, Tran *et al.* [3] compared 14 integration methods across five different integration tasks varying in complexity using four assessment metrics. They recommended Harmony [6], LIGER [7], and Seurat v3 [8] methods based on their overall performance. Meanwhile, Richards *et al.* [4] focused on assessing batch correction specifically for the integration of cancer scRNA-seq data using the LISI (Local Inverse Simpson’s Index) metric. Among the five tools tested, STACAS [9] and fastMNN [10] were identified as the superior methods regarding their ability to batch-correct the data without losing tumour-specific biological variability. A more recent benchmark established by Luecken *et al.* [5] used the most comprehensive set of integration performance metrics to date to compare 16 integration methods across seven scRNA-seq datasets with/without scaling and with/without feature selection. The three best-performing methods overall were scANVI [11], Scanorama [12], and scVI [13]. The lack of agreement in findings using these independent benchmarks demonstrates that there is no single method that works best for all integration tasks. Instead, the most suitable method depends greatly on the aim of the study, the type of data, and the degree of noise introduced by the batch.

Among the methods mentioned above, scANVI and scVI are deep neural network methods that are highly effective in correcting complex batch effects [5], although their interpretability is lower than that of other alternatives. In addition, scANVI requires cell label annotations, which are often absent, whereas scVI performs better with larger numbers of cells and more complex batch correction tasks [5]. In contrast, Scanorama, fastMNN, Seurat v3, and STACAS integrate data through mutual nearest neighbours searches in joint low-dimensional embedding, which enables the removal of technical noise in a less computationally intensive manner [5, 14]. However, the use of a joint low-dimensional representation to integrate the batches may cause cell-type “misalignment” if the cell types are not conserved across batches [3, 14]. LIGER uses integrative non-negative matrix factorization to identify shared factors across batches [7], whereas Harmony is a linear integration method that iteratively applies *K*-means clustering in principal component space [6]. Both of these approaches tend to prioritize batch correction over biological conservation [5], making them less suitable to integrate datasets with nuanced biological cell states.

To overcome many of these limitations, we here introduce Coralysis, an R package featuring a multi-level integration algorithm for sensitive integration, reference-mapping, and cell-state identification in single-cell data. Coralysis performs consistently well across diverse scRNA-seq integration tasks and query-reference scenarios. It outperforms state-of-the-art integration methods in tasks in which batches do not share similar cell types, enables integration of rare cell populations beyond transcriptomics, including single-cell proteomic assays, and achieves accurate reference-mapping of previously misclassified cells. Finally, a key feature of Coralysis is that it provides probability scores to identify transient and steady cell states along with their differential expression programs, providing a comprehensive approach for single-cell data integration.

## Materials and methods

### Iterative clustering projection algorithm: adaptations

A multi-level integration method was implemented in Coralysis by adapting the Iterative Clustering Projection (ICP) algorithm, available in ILoReg, [15] to work in a top-down fashion. In addition, clustering accuracy was improved and biases towards more abundant cell types and batches were effectively corrected. Coralysis is a horizontal integration method that requires, as input, a log-normalized expression matrix (features × cells) of shared features along with the batch identity for each cell. Coralysis is distributed as an R/Bioconductor package and is available on GitHub (https://github.com/elolab/Coralysis) and Bioconductor (https://www.bioconductor.org/packages/release/bioc/html/Coralysis.html). The ICP algorithm is described briefly next as it is at the core of our method.

The ICP algorithm utilizes self-supervised learning and clustering comparison to iteratively cluster the data. Assume that we have a dataset


\begin{eqnarray*}
X = \{ \mathbf{x}_1, \ldots, \mathbf{x}_n \}
\end{eqnarray*}

of *n* cells that we want to partition into *k* disjoint subsets (clusters):


\begin{eqnarray*}{{\pi }_k} = \left\{ {{{C}_1},\ldots,{{C}_k}} \right\}
\end{eqnarray*}

Here, the vector ${\boldsymbol{x}_i} \in {{\mathbb{R}}^m}$ denotes the normalized molecular profile (e.g. gene expression) of cell *i* across *m* genomic features (e.g. genes) that are present in at least one of the cells. The ICP algorithm seeks a clustering ${{\pi }_k}$ that maximizes the adjusted Rand index (ARI) between ${{\pi }_k}$ and its projection ${{\tilde{\pi }}_k}$ defined by logistic regression, involving four steps detailed below.

Step 1 initializes the clustering by partitioning the *n* cells into *k* clusters either randomly (as in ILoReg) or using a structured strategy (see “Cluster initialization with Coralysis”):
  \begin{eqnarray*}\pi _k^{\left( 1 \right)} = \left\{ {C_1^{\left( 1 \right)},\ldots,C_k^{\left( 1 \right)}} \right\}
\end{eqnarray*}

Here $t = 1$ denotes the first epoch, while $\pi _k^{( t )}$ denotes the clustering at epoch *t*:


\begin{eqnarray*}\pi _k^{\left( t \right)} = \left\{ {C_1^{\left( t \right)},\ldots,C_k^{\left( t \right)}} \right\}
\end{eqnarray*}

Step 2 prepares a balanced training dataset $X_{\textit{train}}^{( t )}$ for logistic regression in epoch *t*. The original ICP (as in ILoReg) uses down- or over-sampling to select an equal number of cells from each cluster of $\pi _k^{( t )}$, whereas Coralysis enables the creation of a separate training set (see Section “Separate training set with Coralysis”) and use of information about different cell batches ${{D}_b}$, $ b \in {1,\ldots,B} $, to improve batch balance during training (see Section “Batch balance during Coralysis training”).
Step 3 trains an L1-regularized logistic regression classifier using the training dataset $X_{\textit{train}}^{( t )}$ and the corresponding cluster labels $y_{\textit{train}}^{( t )}$ based on clustering $\pi _k^{( t )}$ using the LiblineaR package [16]. Given a set of pairs $( {{\boldsymbol{x}_i},{{y}_i}} )$ of the input vectors and their labels, LiblineaR implements the L1-regularized logistic regression by solving:
  \begin{eqnarray*}
\underset{\mathbf{w}}{\min} \left\{ \sum_i \log\left(1 + e^{-y_i \mathbf{w}^T \mathbf{x}_i}\right) + \lambda \lVert \mathbf{w} \rVert_1 \right\}
\end{eqnarray*}

where the vector $\boldsymbol{w}$ provides the optimized solution, $\Vert \boldsymbol{w}{{\Vert}_1}$ denotes its 1-norm, and the parameter $\lambda $ controls the balance between regularization and loss. For multi-class analysis, the one-vs-rest strategy is utilized, where binary classifiers are trained for each cluster using a sequential approach. Finally, the full dataset $X$ is projected using the trained classifier to obtain the projected clustering $\tilde{\pi }_k^{( t )}$, where each cell *i* is assigned to the cluster with the highest predicted probability:


\begin{eqnarray*}\tilde{y}_{ki}^{\left( t \right)}\ = \mathop {{\mathrm{\ argmax}}}\limits_{j\ \in \ \left\{ {1,\ldots,k} \right\}} p_{kij}^{\left( t \right)}
\end{eqnarray*}

Here, $p_{kij}^{( t )}$ denotes the estimated probability that cell *i* belongs to cluster *j*, and


\begin{eqnarray*}P_k^{\left( t \right)} = \left[ {p_{kij}^{\left( t \right)}} \right]{{\ }_{n \times k}}
\end{eqnarray*}

is the resulting $n \times k$ probability matrix.

Step 4 determines the agreement between the current clustering $\pi _k^{( t )}$ and its projection $\tilde{\pi }_k^{( t )}$ using the adjusted Rand index:
  \begin{eqnarray*}ARI\left( {\pi _k^{\left( t \right)},\tilde{\pi }_k^{\left( t \right)}} \right)
\end{eqnarray*}

where a value of 1 indicates perfect agreement and a value close to 0 random labels. If the ARI increases over the previous value (initialized as 0 at $t = 1$):


\begin{eqnarray*}ARI\left( {\pi _k^{\left( t \right)},\tilde{\pi }_k^{\left( t \right)}} \right) > ARI\left( {\pi _k^{\left( {t - 1} \right)},\tilde{\pi }_k^{\left( {t - 1} \right)}} \right)
\end{eqnarray*}

then we update $\pi _k^{( {t + 1} )} = $$\tilde{\pi }_k^{( t )}$ and repeat steps 2–4 for it in the new epoch. Otherwise, steps 2–4 are repeated with the same $\pi _k^{( t )}$ until a maximum number of reiterations *r* is reached (default $r = 5$) or a maximum number of iterations $max.\textit{iter}$ is reached (default $max.\textit{iter} = 200$).

The final output of ICP is the clustering ${{\pi }_k} = \pi _k^{( T )}$ from the last epoch *T* and the corresponding probability matrix ${{P}_k} = P_k^{( T )} = [ {{{p}_{kij}}} ]{{\ }_{n \times k}}$. To ensure robustness, ICP is repeated *L* times using independent runs with different random seeds (by default $L = 50$). The adaptations introduced to the ICP algorithm will be described in the sections below.

### The divisive ICP algorithm

To enable clustering in a top-down fashion by sequentially increasing the number of clusters *k*, Coralysis runs ICP iteratively over multiple rounds *q*, doubling the number of clusters in each round, i.e. $k = {{2}^q}$, until the target number of clusters is reached (by default $K = 16$). In round $q = 1$, ICP is applied to obtain an initial clustering into two clusters:


\begin{eqnarray*}{{\pi }_2} = \left\{ {{{C}_1},{{C}_2}} \right\}
\end{eqnarray*}

In each following round $q > 1$, each cluster from the previous round $q - 1$ is split into two based on batch-wise cluster probability maxima of the cells to initiate ICP. Let $p_{ki}^{max}$ denote the highest cluster assignment probability for cell *i* across the clusters, where $k = {{2}^{q - 1}}$:


\begin{eqnarray*}p_{ki}^{max} = \mathop {\max }\limits_{j\ \in \ \left\{ {1,\ldots,k} \right\}} {{p}_{kij}}
\end{eqnarray*}

and let ${{\tau }_{jb}}$ denote the batch-specific median of these maximum assignment probabilities for cells in cluster *j* and batch *b*:


\begin{eqnarray*}{{\tau }_{jb}} = \mathop {{\mathrm{median}}}\limits_{i\in {{C}_j} \cap {{D}_b}} \left\{ {p_{ki}^{max}} \right\}
\end{eqnarray*}

Using these batch-specific medians as thresholds, each cluster ${{{C}}_{j}}$ of clustering ${{{\pi}}_{k}}$ is divided into two subclusters, one consisting of cells with lower assignment probabilities ${{{C}}_{{j}1}}$ and the other with higher probabilities ${C}_{j2}$:


\begin{eqnarray*}
C_{j1} = \bigcup_{b=1}^{B} \left\{\, i \in C_j \cap D_b \;\middle|\; p_{ki}^{\max} \le \tau_{jb} \right\}
\end{eqnarray*}

\begin{eqnarray*}
{C}_{{j}2} = \bigcup_{b=1}^{B} \left\lbrace {i}\in{C}_{j} \cap {D}_{b} | {p}_{ki}^{max} > {\tau}_{jb} \right\rbrace
\end{eqnarray*}

ICP is then applied using this refined clustering as a starting point for the round to obtain clustering with ${k} = {{2}^{q}}$ clusters. This process is repeated iteratively until the target number of clusters *K* is reached.

### Cluster initialization with Coralysis

Coralysis initializes the divisive ICP by partitioning the training data ${X}_{{train}}^{( 1 )}$ into two starting clusters in a batch-wise manner based on the first principal component (PC1). Principal component analysis is performed using the irlba package [17] on standardized (*Z*-scored) data, using all cells from all batches and all features with non-zero variance. Let ${s}_{i}^{{PC}1}$ denote the PC1 score of cell *i* and ${{\eta }_b}$ the batch-specific median PC1 score for batch *b*:


\begin{eqnarray*}{{\eta }_b} = \mathop {{\mathrm{median}}}\limits_{i\in{{D}_b}} \left\{ {s_i^{PC1}} \right\}
\end{eqnarray*}

Cells are assigned to one of the initial clusters according to their batch-specific median PC1 score:


\begin{eqnarray*}
C_1^{\left( 1 \right)} = \bigcup_{b=1}^{B} \left\{ {i\in{{D}_b}|\ s_i^{PC1}\ \le {{\eta }_b}} \right\}
\end{eqnarray*}

\begin{eqnarray*}
C_2^{( 1)} = \bigcup_{b=1}^{B} \left\{ {i\in{{D}_b}| {\ s_i^{PC1}\ } > {{\eta }_b}} \right\}
\end{eqnarray*}

This initial clustering is used in the first epoch $t = 1$ of ICP. This approach reduces the algorithm runtime, as it takes less epochs to converge, and it favours integration due to the more concordant clusterings obtained across *L* ICP runs. The aim of the initial clustering approach is to provide a rough, meaningful clustering result that is not robust, allowing the ICP algorithm to iterate over the clusters and converge.

### Separate training set with Coralysis

To avoid using the same data for training and testing and to provide a more balanced and less sparse representation of the cells, Coralysis constructs a separate training set $X_{\textit{train}}^{( 1 )}$. First, highly variable features of the full data matrix $X$ are identified using the scran R package [18], with a default of 2000 features. Second, a principal component analysis is performed on these features using the irlba package [17], retaining the top 30 principal components by default. These are then used to divide the cells into a large number of groups ${{G}_a}$ (default $500$) using *k*-means++ from the flexclust package [19]. Finally, the average profile of cells in each group is calculated:


\begin{eqnarray*}
\bar{\mathbf{x}}_{a} = \frac{1}{\lvert G_a \rvert} \sum_{i \in G_a} \mathbf{x}_{\!i}
\end{eqnarray*}

where $| {{{G}_a}} |$ denotes the number of cells in group *a*.

If batch labels are available, this procedure is applied separately for each batch and the resulting batch-wise training sets are concatenated into a single training matrix:


\begin{eqnarray*}
X_{\textit{train}}^{\left( 1 \right)} = \left\{ {{{{ \boldsymbol{\bar{x}}}_1} },\ldots,{{{ \boldsymbol{\bar{x}}}_A} }} \right\}
\end{eqnarray*}

where *A* denotes the total number of average profiles across the batches. For clarity, these are also referred to as cells in the method description.

### Batch balance during Coralysis training

Assuming that the most confidently assigned cells are central to the different clusters across batches, at each epoch $t > 1$, Coralysis identifies for each cluster *j* and each batch *b*, the representative cell with the highest assignment probability from the current clustering $\pi _k^{( t )}$ and the associated probability matrix $P_k^{( t )}$:


\begin{eqnarray*}
i_{jb}^{(t)}\ = \mathop{{\mathrm{argmax}}}\limits_{i \in C_j^{(t)} \cap {{D}_b}} p_{kij}^{(t)}
\end{eqnarray*}

The *k*-nearest neighbours of each selected cell $i_{jb}^{( t )}$ are then retrieved using the RANN package [20]. The number of neighbours can be specified as fixed or proportional to the size of the clusters. By default, we set it to be proportional to the size of each cluster *j*:


\begin{eqnarray*}
\left\lfloor \frac{{0.3 \cdot \left| {C_j^{\left( t \right)}} \right|}}{B} \right\rfloor
\end{eqnarray*}

The selected cells across clusters and batches are then used to form the refined training dataset $X_{\textit{train}}^{( t )}$, where inclusion of neighbours from different batches enables improving batch balance.

This selection procedure aims to find the most similar cells across batches by using probability as a proxy for similarity and to achieve a more balanced set of cells for training. Additionally, it contributes to the faster convergence of the ICP algorithm by relying on the neighbours of the cells with the highest probabilities.

### Coralysis reference-mapping method

The Coralysis reference-mapping method enables projection of unannotated query datasets onto annotated reference datasets with known cell types using solely the ICP framework. This allows efficiently transferring cell type labels to new data without the need to retrain ICP on the query data, relying on the already trained ICP models. The procedure begins by running the original or divisive ICP *L* times on a reference dataset that contains known cell type annotation labels. Cluster assignment probabilities across the runs are obtained and used to perform principal component analysis. For each cell in the query dataset, the trained ICP models are then used to calculate cluster probability vectors $\boldsymbol{p_l^{\textit{query}}} \in{{\mathbb{R}}^k}$ across each run *l*, which are projected onto the annotated reference PCA space performed with the stats package [21]. A *k*-nearest neighbours classifier is trained on the reference PCA scores and their known labels using the class R package [22]. Each query cell is classified by identifying its *k* -nearest neighbours in the reference space (default $k.nn = 10$) and the predicted label is assigned by majority voting among the neighbours. Additionally, the proportion of neighbours supporting the winning class is recorded as a confidence score for the prediction.

### Benchmarking integration: scib-pipeline

Coralysis integration method was benchmarked against six unsupervised integration methods (scVI, Scanorama, Seurat v4 CCA/RPCA, Harmony, and fastMNN) across six datasets (pancreas, lung, human immune, human/mouse immune, and two simulations) using 14 assessment metrics within the publicly available scib-pipeline (https://github.com/theislab/scib-pipeline) [5]. The mouse brain dataset was not included in the benchmark because it was not available on figshare with the other datasets due to size limitations. The pancreas integration task comprised six human pancreas scRNA-seq datasets, each generated with a different technology (CEL-seq, CEL-seq2, Fluidigm C1, inDrop, SMARTer, and SMART-seq2), with datasets treated as batches (or four donors as batches for inDrop—nine batches in total). The lung task consisted of three healthy human lung scRNA-seq datasets generated using Drop-seq and 10X Chromium from distinct spatial locations (parenchyma in lung transplants and airways in biopsies), with 16 donor samples treated as batches. The human immune task included scRNA-seq of immune cells from five studies, two tissues (bone marrow and peripheral blood), two technologies (10X Chromium and SMART-seq2), and 10 samples treated as batches. The cross-species immune task combined these 10 human samples with 13 mouse samples (some using Microwell-seq), totaling 23 samples treated as batches. All datasets with available counts were normalized using scran pooling, except those only available in RPKM or TPM, and all were log-transformed with pseudocount of 1. The two simulation datasets were generated with the Splat model from Splatter: Simulation 1 included six batches with controlled differences in cell type proportions and library sizes, while Simulation 2 simulated nested batch effects by adding batch-specific noise, yielding 16 sub-batches. A detailed description of the datasets can be found in Luecken *et al.* [5], where the datasets were originally generated and preprocessed. The version of R and Seurat was updated to 4.1.3 and 4.3, respectively. The forked version of the *theislab/scib-pipeline* github repository with the adaptations required to reproduce the benchmark is available at https://github.com/elolab/scib-pipeline. The overall score was calculated as a weighted mean of batch correction and biological conservation metrics, with respective weights of 0.4 and 0.6. Methods that failed to run for a particular task were removed. The benchmark was run with Snakemake (v.7.25.2) [23] in a cluster environment with Slurm (v.23.02.6) with 8 threads and 354 GB of RAM. The integration performance was summarized and visualized using custom R functions used in Luecken *et al.* [5] and available at https://github.com/theislab/scib-reproducibility/tree/main/visualization.

### Accuracy of reference-mapping across query-reference scenarios

The accuracy of the Coralysis reference-mapping method was assessed across four different query-reference scenarios: (i) imbalanced cell types, (ii) unshared cell types, (iii) unrepresented batch, and (iv) a varied strength of the batch effect. Performance was measured in terms of accuracy as follows:


\begin{eqnarray*}\textit{Accuracy} = \frac{{TP + TN}}{{TP + TN + FP + FN}},
\end{eqnarray*}

with $TP = $ True Positives, $TN = $ True Negatives, $FP = $ False Positives, $FN = $ False Negatives. These analyses were performed with the R programming language in a containerized docker image publicly available at Docker Hub (*elolabfi/sctoolkit*) running R (v.4.2.1) [21] and RStudio server (2022.07.2 Build 576).

### Imbalanced cell types

Two scRNA-seq datasets (batches 1 and 2) of peripheral blood mononuclear cells (PBMCs) were used to assess the accuracy of reference-mapping for a scenario when the reference has imbalanced cell types relative to the query. The batch 1 dataset was used as reference and the batch 2 as query. Each dataset was comprised of six balanced cell types (B cell, CD4 T cell, CD8 T cell, Monocyte_CD14, Monocyte_FCGR3A, and NK cell), i.e. each cell type had the same number of cells (*n* = 200). In total, each dataset had 1 200 cells and 33 694 genes. The analyzed PBMC datasets are from Maan *et al.* [24], and they can be downloaded at figshare: https://doi.org/10.6084/m9.figshare.24625302.v1. Both datasets were normalized by applying the natural-log transformation to the gene expression counts, divided by the total counts per cell multiplied by a scaling factor (10 000). The reference-mapping was performed by randomly downsampling one cell type in the reference to 10%; training the reference with ICP (2000 highly variable genes [HVGs]); and projecting and classifying the query against the reference. This procedure was repeated independently for every cell type in the reference.

### Unshared cell types

The two PBMC datasets used for the imbalanced cell type query-reference scenario were used for assessing the performance of the reference-mapping when the reference has unshared cell types relative to the query. The reference-mapping analysis followed the same procedure described in Section “Imbalanced cell types” but replacing the downsampling by ablation, i.e. completely removing one cell type at a time from the reference.

### Unrepresented batch

A set of pancreatic scRNA-seq datasets comprising eight samples (celseq, celseq2, smartseq2, fluidigmc1, indrop1, indrop2, indrop3, and indrop4) sequenced across five library preparation technologies (SMARTSeq2, Fluidigm C1, CelSeq, CelSeq2, and inDrops) was used to assess the performance of reference-mapping with and without shared batch effects. Every sample corresponded to a pancreatic scRNA-seq dataset sequenced using a different library preparation technology with the exception of the samples indrop1, indrop2, indrop3, and indrop4, which corresponded to four biological replicates coming from the same technology inDrops. The datasets were normalized as described in Section “Imbalanced cell types” and genes not expressed were removed. In total, the datasets comprised 14 890 cells and 29 340 expressed genes. The number of cells per sample varied from 638 in fluidigmc1 to 3605 in indrop3 (celseq =  1004; indrop4 = 1303; indrop2 = 1724; indrop1 = 1937; celseq2 = 2285; smartseq2 = 2394). A reference was built by integrating six samples, celseq, celseq2, fluidigmc1, indrop1, indrop3, and indrop4, using Coralysis with 2000 HVGs. The samples indrop2 and smartseq2 were chosen as query datasets representing a scenario of a shared and unshared batch effect between the reference-query, respectively. Then the query datasets were classified and projected onto the integrated reference with Coralysis. The pancreatic datasets used herein were obtained from the SeuratData package (v.0.2.1).

### A varied strength of the batch effect

Two human PBMC scRNA-seq samples representing resting and interferon-stimulated cells were used for assessing the performance of reference-mapping when the gene expression of different cell types within a sample respond distinctly to a batch effect or a biological condition, i.e. the transcriptome changes more for some cell types than others. The resting sample was chosen as reference (6548 cells) and the interferon-stimulated sample as query (7451). The datasets were normalized as described in Section “Imbalanced cell types” and genes not expressed were removed. In total, the samples comprised 14 044 genes and 13 999 cells. The reference was trained using Coralysis with 2000 HVGs and the query classified and projected onto the reference. The PBMC data used for this task were obtained from the SeuratData package (v.0.2.1).

### Assessing the impact of switching the reference and query datasets on accuracy

The three datasets used for assessing the “Accuracy of reference-mapping across query-reference scenarios” were used for assessing the impact of switching the reference and query datasets on accuracy. The pancreas datasets generated with indrop2 and smartseq2 technologies were used to assess the impact of switching the reference and query datasets when these represent a batch effect. The resting and interferon-stimulated PBMC datasets were used to assess the impact of switching the reference and query when these represent different biological conditions and the two additional batches of PBMCs for assessing the impact of cell type imbalance. Each completely balanced cell type from the two batches of PBMCs was downsampled in either batch to 0% (complete absence), 5%, 10%, 25%, 50%, and 100% (fully balanced) in order to simulate cell type imbalance. For each cell type imbalance dataset, the non-expressing genes were removed and the data was normalized similarly as in Section “Imbalanced cell types”.

Every reference was trained through ICP using 2000 HVGs with default parameters, as described in Section “Imbalanced cell types”. The UMAPs for the references were computed with the uwot method with the custom parameters: *min_dist = 0.3* and *n_neighbours = 30*.

The replicability experiment was performed for the resting and interferon-stimulated PBMC datasets. It was performed ten reference-mapping experiments using either resting (CTRL) or interferon-stimulated (STIM) PBMCs as the reference, under three different settings: 2000 HVGs and L1-regularization, 6000 HVGs and L1-regularization, and 2000 HVGs and L2-regularization. For each reference-mapping experiment, each cell type from each one of the datasets was randomly sampled to 70% to ensure variability across the experiments. The coefficients were retrieved for each reference dataset by providing the ground-truth cell type labels to the *MajorityVotingFeatures* function in Coralysis, which finds the best matching ICP cluster for every cell type label through a majority voting approach and retrieves the respective coefficients.

### Integration of PBMCs from two 10x 3′ assays

Two 10X scRNA-seq 3′ assays—V1 and V2—from human PBMCs were integrated with Coralysis to demonstrate multi-level integration in practice. Both assays were downloaded from the 10X website: V1 (https://support.10xgenomics.com/single-cell-gene-expression/datasets/1.1.0/pbmc6k) and V2 (https://support.10xgenomics.com/single-cell-gene-expression/datasets/2.1.0/pbmc8k). Cells were annotated using the annotations described in the file “Source Data Fig. 4” from Korsunsky *et al.* [6]. Cells without annotation were discarded (143 cells). Duplicated gene symbols were renamed by appending the corresponding Ensembl IDs. Genes not expressed were removed. In total 20 016 genes and 13 016 cells remained—4770 cells in V1 and 8276 cells in V2. Normalization was done similarly as in Section “Imbalanced cell types”. Highly variable genes (HVGs = 2000) were selected with the package scran. The two assays were integrated using Coralysis by running the function *RunParallelDivisiveICP*, using 2000 HVGs and default parameters, except for *allow.free.k = FALSE, C = 1, train.k.nn = 0.45*, and *ari.cutoff = 0.1*, which were specified to ensure that Coralysis returned exactly 16 clusters. By default, *allow.free.k = TRUE*, allowing clusters without assigned cells to be dropped among the target number of clusters (default 16). Setting *allow.free.k = FALSE* overrides this behaviour to enforce the return of all 16 clusters with the support of more relaxed parameters: *C = 1, train.k.nn = 0.45*, and *ari.cutoff = 0.1*. The probability tables for each final divisive round at *K* = 2, *K* = 4, *K* = 8, and *K* = 16 were independently concatenated across the 50 ICP runs to compute a PCA and t-distributed Stochastic Neighbour Embedding (t-SNE) with Coralysis. Probabilities were centered and scaled before PCA. The divisive ICP clustering run number two (*l* = 2) corresponding to the cluster probability table (at K16) with the highest standard deviation was selected to exemplify the evolution of batch label mixing and cell type separation across one divisive ICP run. The top ten positive coefficients for every cluster across the four divisive ICP rounds for the run number 2 were also retrieved. The t-SNE and cluster tree plots were made with ggplot2 (v.3.5.1) [25], pie charts with scatterpie (v.0.2.3) [26], and heatmaps with ComplexHeatmap (v.2.14.0) [27].

### Integrating similar unshared cell type pairs

Two PBMC scRNA-seq samples, one representing resting PBMCs (CTRL) and other PBMCs stimulated with interferon (STIM), were used to highlight the ability of Coralysis to integrate similar unshared cell type pairs across batches. The *ifnb* dataset (v.3.1.0) published by Kang *et al.* [28] was obtained from the SeuratData R package (v.0.2.1). Cells were normalized as described in Section “Imbalanced cell types” and genes not expressed were removed. Two pairs of similar cell types that responded differently to interferon stimulation were selected: CD14–CD16 monocytes and CD4 naive–memory T cells. For each selected cell type pair, one cell type was retained in one sample and removed from the other to simulate a situation where batches do not share similar cell types. Specifically, CD16 monocytes (507 cells) and CD4 memory T cells (859) were retained in the CTRL sample but removed from STIM, while CD14 monocytes (2147) and CD4 naive T cells (1526) were retained in STIM but removed from CTRL. In total 14 044 genes and 9366 cells remained (3355 cells in CTRL and 6011 in STIM). Finally, the integration task was run through the scib-pipeline as described in Section “Benchmarking integration: scib-pipeline”. The *stim* and *seurat_annotations* cell metadata columns were used as batch and cell-type labels, respectively.

### Benchmarking methods for imbalanced cell type integration

The two PBMC datasets used for the imbalanced and unshared cell type query-reference scenarios were used for benchmarking cell type imbalance integration with the scib-pipeline. B cells, CD14 monocytes, and FCGR3A monocytes were selected to assess the impact of cell type imbalance on integration performance. Each of these cell types was downsampled in batch “batch_2” to 0% (complete absence), 5%, 10%, and 100% (fully balanced, i.e. 200 cells per cell type in each batch), resulting in ten distinct datasets. Genes with ≤10 counts were removed and the data was normalized similarly as in Section “Imbalanced cell types”. The overall score was calculated as a weighted mean of batch correction and biological conservation metrics, with respective weights of 0.4 and 0.6. Methods that failed to run for a particular task were excluded, and the remaining methods were ranked based on their average rank across the ten datasets. The benchmark was run with Snakemake (v.7.25.2) [23] in a cluster environment with Slurm (v.23.02.6) with 100 threads and 384 GB of RAM. The integration performance was summarized and visualized using custom R functions used in Luecken *et al.* [5] and available at https://github.com/theislab/scib-reproducibility/tree/main/visualization.

### Integration of single-cell proteomics assays

Two types of single-cell proteomics assays were integrated with Coralysis: antibody-derived tags (ADTs) and cytometry by time of flight (CyTOF). The datasets were obtained from https://github.com/single-cell-proteomic/SCPRO-HI [29]. The cell-type annotations used were the same as given in Koca and Sevilgen [29]. The ADT data consisted of human PBMCs from eight HIV-infected donors (P1–8) following their longitudinal response across vaccinated and unvaccinated individuals (published by Hao *et al.* [30]). Batch 1 comprised donors P1–4, and batch 2 donors P5–8. In total, the ADT dataset included 228 proteins and 161 764 cells (67 090 and 94 674 cells in batches 1 and 2, respectively). Cells were transformed using centered log-ratio (CLR) normalization prior to integration, utilizing the *NormalizeData* function from Seurat. The batch and cell-type labels used were *Batch* and *cluster_s*, respectively. The *celltype.l2* labels originally given in Hao *et al.* [30] were also highlighted.

The CyTOF data consisted of human whole blood datasets from Rahil *et al.* [31] and Bjornson-Hooper *et al.* [32]. The dataset from Rahil *et al.* [31] included whole blood from 35 donors infected with H1N1 influenza across 11 timepoints, while the dataset from Bjornson-Hooper *et al.* [32] comprised blood samples from healthy donors exposed *in vitro* to 15 different stimuli. Only the data corresponding to the interferon gamma (IFN$\gamma $) stimulus was used for the latter. The dataset integrated included the expression of 39 proteins across 216 322 cells (102 147 cells from *h1n1* dataset, and 114 175 cells from *ifng* dataset). The two datasets were considered as batches and the *cluster_s* cell metadata column as the cell type label. The dataset was scaled (*Z*-score) by proteins before integration. The first seven principal components were used for building the training set instead of the default 30 due to the low number of features (*n* = 39).

In both integration tasks, all features were used for integration and the resulting probabilities were centered and scaled before PCA with Coralysis. In addition, the fast implemented version of UMAP available in the uwot (v.0.1.14) [33] R package was used to compute UMAP. The Python package scib-metrics [5] was used to compute batch correction and bio-conservation metrics without the calculation of isolated labels as the batches shared all cell types.

### Mapping PBMCs from different sequencing technologies

The *pbmcsca* scRNA-seq dataset (v.3.0.0) from SeuratData was used to quantify the mapping accuracy performance of Coralysis. The dataset published by Ding *et al.* [34] comprised nine PBMC samples prepared with different single-cell library preparation technologies: 10x Chromium (v2) A, 10x Chromium (v2), 10x Chromium (v2) B, 10x Chromium (v3), CEL-Seq2, Drop-seq, inDrops, Seq-Well, and Smart-seq2. The sample corresponding to PBMCs obtained through 10x Chromium (v2) A (3222 cells) was selected as a reference and the remaining as query datasets (27 753 cells). Cells without annotation, i.e. with label *Unassigned* in the *CellType* cell metadata column, were removed. The data were normalized as described in Section “Imbalanced cell types” and genes not expressed were discarded. The top 2000 most HVGs were selected with the scran function *modelGeneVar* for the reference sample (10x Chromium (v2) A). The reference sample was trained with Coralysis function *RunParallelDivisiveICP*, with default parameters, with the exception of *divisive.method = “cluster”*, as the reference sample did not have cells originating from different batches, i.e. *batch.label = NULL*. The PCA and UMAP were computed with Coralysis with the argument *return.model = TRUE*. Probabilities were scaled prior to PCA as described in Section “Integration of PBMCs from two 10x 3′ assays”. Reference-mapping was computed with the Coralysis function *ReferenceMapping* with default parameters and cells were projected onto the reference UMAP (*project.umap = TRUE*). Visualizations were obtained with Coralysis, ggplot2 (v.3.5.1), and scater (v.1.26.1) [35].

### Inference of cell states with cell cluster probabilities

CD34+ haematopoietic stem cells (HSCs) from Persad *et al.* [36] were used to show how Coralysis cell cluster probability can be biologically meaningful. The scRNA-seq dataset consisted of 6881 cells downloaded from Zenodo: https://zenodo.org/records/6383269/files/cd34_multiome_rna.h5ad. The top 2000 HVGs were selected with the scran function *getTopHVGs* before running Coralysis function *RunParallelDivisiveICP* with *divisive.method = “cluster”*. UMAP was calculated with the uwot package and parameters were adjusted to provide more connectivity: *n_neighbours = 100, min_dist = 0.5*. The *palantir_pseudotime* [37] variable was used to compute the Pearson coefficient with the mean cell cluster probability obtained with Coralysis across 50 ICP runs. The cell type labels used corresponded to those available in the *celltype* variable as used in the original study.

Human embryoid body cells (31 029 cells) from Moon *et al.* [38] were integrated by embryonic stage (0–1, 2–3, 4–5, 6–7, and 8–9) with Coralysis using 6000 HVGs. UMAP was computed as described above for the CD34+ cells dataset. The Coralysis cell cluster probabilities were divided into 20 evenly sized bins for each cell type independently. Gene expression and cell cluster probabilities were then averaged within each bin for each cell type to calculate the Pearson coefficient between them.

Visualizations were obtained with ggplot2 (v.3.5.1) and ComplexHeatmap (v.2.14.0).

### Code availability

The multi-level integration and reference-mapping methods have been implemented into the R/Bioconductor package Coralysis publicly available on GitHub (https://github.com/elolab/Coralysis) and Bioconductor (https://www.bioconductor.org/packages/release/bioc/html/Coralysis.html). The forked version of the *theislab/scib-pipeline* github repository with the adaptations required to reproduce the benchmark described herein is available at https://github.com/elolab/scib-pipeline. Finally, all the R and Python scripts required to reproduce the remaining analyses and figures are available at https://github.com/elolab/Coralysis-reproducibility. The analyses can be reproduced using the Coralysis version corresponding to commit *47f1b3415663ee895df188f264ac4d8ad8d24c11*, which can be installed via *devtools::install_github(“elolab/Coralysis”, ref = “47f1b3415663ee895df188f264ac4d8ad8d24c11”)*.

## Results

### Coralysis identifies cell types through multi-level integration

**Figure 1.**
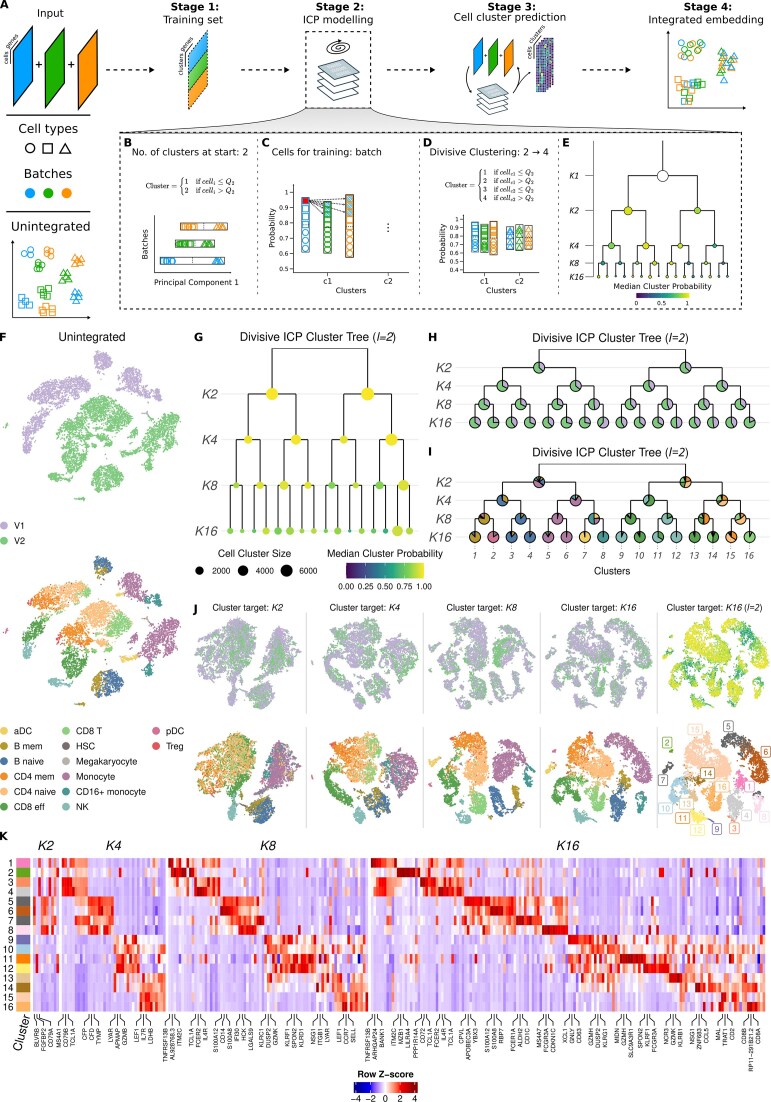
Demonstration of Coralysis multi-level integration method. (**A**) Coralysis utilizes self-supervised learning to iteratively cluster heterogeneous single-cell datasets across batches (shown here in blue, green, and orange). After constructing a batch-wise balanced training set, the ICP algorithm is applied to iteratively maximize the agreement between the clustering and its projection using regularized logistic regression. This yields cluster assignment probabilities for cell cluster prediction, which are then used to construct an integrated embedding. Several adaptations were made to our original ICP algorithm [15] to enable a top-down, divisive strategy that sequentially increases the number of clusters while integrating across batches. (**B**) Coralysis initializes the divisive ICP by partitioning the data into two clusters in a batch-wise manner based on the first principal component (PC1), using batch-wise median as a threshold. (**C**) At each subsequent epoch, Coralysis refines the training set by identifying the most confidently assigned cells per cluster and batch and their *k*-nearest neighbours to ensure batch balance during training. (**D**) After convergence, each cluster is split into two based on batch-specific medians of maximum cluster assignment probabilities. (**E**) This process is repeated in multiple divisive ICP clustering rounds, enabling multi-level integration that reduces batch effects and progressively enhances biological resolution until the target number of *K* clusters is reached. (**F**) Unintegrated PBMCs from two 10x 3′ assays, V1 (*n* = 4770 cells) and V2 (*n* = 8276 cells), projected onto t-SNE comprising aDCs, B memory cells (B memory), B naive cells, CD4 memory cells (CD4 mem), CD4 naive cells, CD8 effector T cells (CD8 eff), CD8 T cells (CD8 T), HSCs, megakaryocytes, monocytes, CD16+ monocytes, natural killer cells (NK), plasmacytoid dendritic cells (pDC), and regulatory T cells (Treg). (**G**) Divisive ICP clustering tree for the divisive ICP run corresponding to the K16 cluster probability table with the highest standard deviation (*l* = 2). The size of the dots represents cluster size, and the colour represents the median cluster probability. (**H**) Cell distribution of batches per cluster for divisive ICP run 2. (**I**) Distribution of cell types per cluster for divisive ICP run 2. (**J**) t-SNE projections demonstrating multi-level integration in practice, highlighting the fading of the batch effect and the separation of cell types along the four divisive ICP rounds (2, 4, 8, and 16). (**K**) Top ten positive coefficients for every cluster across the four divisive ICP rounds for run 2. Rows represent the 16 clusters found at divisive ICP run 2 and columns coefficients (i.e. genes). The normalized gene expression data were averaged across the clusters and standardized (*Z*-score) cluster-wise. The top three coefficients for every cluster at every round are labelled.

Coralysis identifies shared cell subpopulations (cell types or states) across a given set of heterogeneous single-cell datasets through four main stages (see Fig. 1A). The first stage clusters the cells batch-wise in a low-dimensional space (PCA) using Kmeans++ [19] with a large number of clusters (default 500). These clusters are used to average the feature expression in order to build a training set. The second stage uses the built training set to identify shared cell clusters across datasets through divisive ICP modelling. In the third stage, the cluster probability is predicted for every cell against every ICP model. Finally, the fourth stage concatenates the cluster probability matrices and performs a PCA to determine an integrated embedding, which can be used for downstream clustering or non-linear dimensionality reduction techniques.

The original ICP algorithm was not designed for clustering heterogeneous scRNA-seq datasets; so, it tends to suffer from technical noise or batch effects, despite the implemented ensemble clustering approach. Therefore, we here adapted the ICP algorithm to perform divisive clustering rounds in order to promote batch label mixing and cell-type separation (Fig. 1B–E). Briefly, we begin with two clusters determined by the distribution of cells along the first principal component, in a batch-wise manner (Fig. 1B). For every epoch (i.e. a single model iteration through the training set), the ICP algorithm selects for training the cells that represent the batch-wise *k*-nearest neighbours with the highest probability (Fig. 1C). Once the algorithm converges to stable clustering, the next clustering round starts by splitting every obtained cluster in two based on the batch-wise cluster probability distribution (Fig. 1D). This process (Fig. 1C and D) is repeated until the algorithm reaches the last clustering round (by default clustering round 4, corresponding to 16 target clusters; Fig. 1E).

To demonstrate how the Coralysis multi-level integration works in practice, we used two 10x scRNA-seq datasets of PBMCs obtained with two different 3′ assays (V1 and V2). The dataset V2 comprised $ \approx $1.7 times more cells than V1 (8276 versus 4770 cells), making this an imbalanced integration task, which is typically more challenging (Fig. 1F). The divisive ICP clustering run number 2 (*l* = 2) corresponding to the cluster probability table (at K16) with the highest standard deviation was selected to exemplify the evolution of batch label mixing and cell-type separation across one divisive ICP run (the algorithm was run 50 times). As expected, the median cluster probability decreased throughout the divisive ICP clustering rounds (i.e. $K2\rightarrow K4\rightarrow K8\rightarrow K16$) (Fig. 1G). The batch label distribution per cluster was dominated by cells coming from the V2 assay, as expected, given that it comprised almost twice as many cells as V1 (Fig. 1H). The only exception was cluster 8, which consisted mainly of CD16+ monocytes at K16 (Fig. 1I). Interestingly, CD16+ monocytes were the only cell type with more cells in the V1 dataset than in the V2 dataset (332 versus 225, respectively). The successful grouping of CD16+ monocytes highlights the ability of Coralysis to handle the integration of imbalanced datasets and cell types.

The clusters at K2 separated monocytes, dendritic cells (DCs), natural killer (NK) cells, and B cells from T cells (Fig. 1I). This separation was also observed in the t-SNE projection of the concatenated cluster probability matrices at K2 across the 50 independent ICP runs (Fig. 1J). A t-SNE projection was used to better highlight the local structure of the data, aiming to more clearly illustrate the mixing of batch labels and the separation of cell types. Notably, the expression of the top three positive gene coefficients for the ICP model corresponded to *BLVRB, FGFBP2*, and *CD79A*, confined to monocytes/DCs, NK cells, and B cells, respectively (Fig. 1K, and Supplementary Fig. S1A and Supplementary Table S1).

At K4, the first cluster gives rise to a B cell (naive and memory) and a monocyte/DC cluster (CD14+ and CD16+ monocytes and activated DCs) (Fig. 1I–K). The second cluster is partitioned into an effector CD8/NK and a CD8/CD4 (naive and memory) T-cell cluster. This division is supported by coefficients of the ICP model, including well-known cell-type-specific genes for B cells (*MS4A1, CD79B*/*A, TCL1A, LINC00926*, and HLA class II histocompatibility antigen genes); monocytes/DCs (*CFP, CFD, TYMP, AIF1, FCN1, S100A8, SAT1, LST1, CST3*, and *CSTA*); effector CD8/NK cells (*LYAR, APMAP*, granzymes [*GZMB*/*H*/*M*], *FGFBP2, CMC1*, chemokines [*CCL5, XCL2*], and *CTSW*); and CD4/8 naive/memory cells (*LEF1, IL7R, LDHB, NOSIP, LDLRAP1, LTB, NPM1, SPOCK2, IL32*, and *CD3D*) (Fig. 1K, and Supplementary Fig. S1B and Supplementary Table S1).

At K8, B memory cells and plasmacytoid DCs separated from B naive cells, and similarly CD14 + monocytes separated from CD16 + monocytes and activated dendritic cells (aDCs) (Fig. 1I–K, and Supplementary Fig. S1C and Supplementary Table S1). The other four clusters involved mostly CD8 effector cells, NK cells, CD4 memory cells, and CD4 naive cells. At K16, the clusters were further divided via the separation of B memory cells (*MS4A1* and *CD79A*/*B*) from pDCs (*IRF7, XBP1*, and *LILRA4*) and aDCs (*FCER1A* and *CD1C*) from CD16+ monocytes (*FCGR3A*), despite the uneven numbers of cells comprising these cell types (Fig. 1I–K, and Supplementary Fig. S1D and Supplementary Table S1). Transcriptionally similar cell types were also further separated, such as NK cells (*GNLY, KLRD1, GZMK*, and *CD63*) from CD8 effector cells (*GZMH*/*K*/*M, KLRG1*, and *CCL5, CST7*). These results demonstrate the sensitivity of Coralysis in integrating cell populations by selecting low-to-high-resolution cell-type-specific gene coefficients along the top-down clustering process.

### Coralysis prioritizes bio-conservation

**Figure 2.**
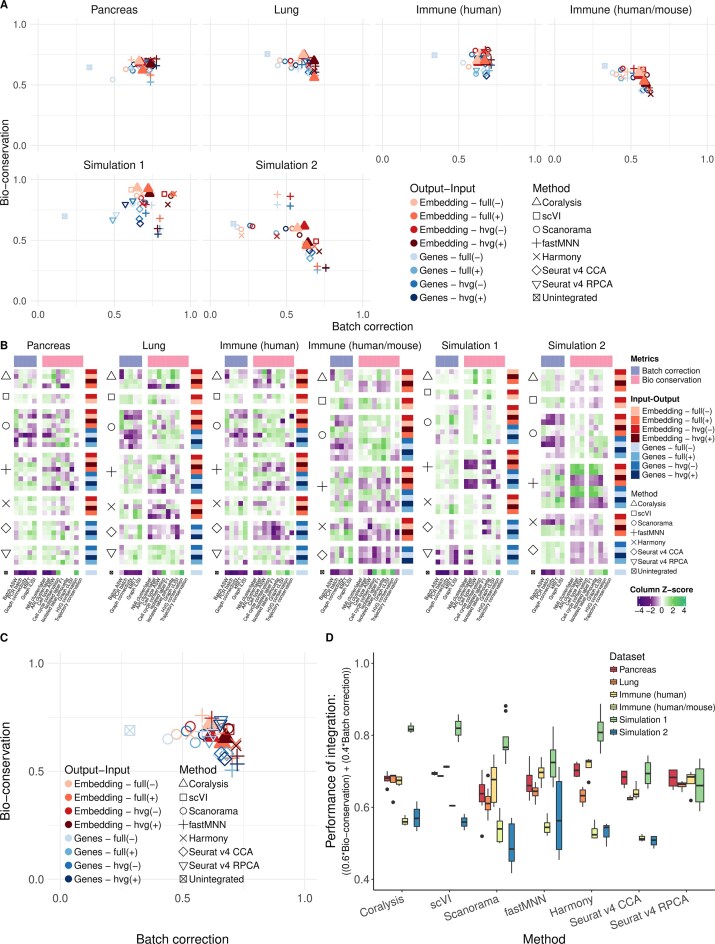
Benchmark of Coralysis integration method through the scib-pipeline. (**A**) Mean of batch-correction (*n* = 5) versus bio-conservation (*n* = 9) scib metrics for each method benchmarked (Coralysis, scVI, Scanorama, fastMNN, Harmony, and Seurat v4 CCA/RPCA) across four real datasets (pancreas, lung, human immune, and human/mouse immune) and two simulated datasets (simulations 1 and 2). Colours represent different output-input and shapes represent distinct methods. Different types of outputs consist of an integrated embedding (embedding) or a batch-corrected gene expression matrix (genes). The different inputs included providing the gene expression data scaled (+) or unscaled (–) and with (hvg) or without (full) feature selection. (**B**) Individual bio-conservation and batch-correction scib metrics for each method and dataset benchmarked. Metrics were standardized (*Z*-score) across methods. (**C**) Averaged bio-conservation and batch-correction scores across the six datasets. (**D**) Variance in performance of integration for different input–output across methods. Performance score consists of the weighted average of bio-conservation (0.6) and batch-correction (0.4) metrics.

We benchmarked Coralysis using the publicly available scib-pipeline [5] in order to perform an independent and unbiased comparison. The top five unsupervised integration methods from the benchmark conducted by Luecken *et al.* [5] were selected for this comparison (scVI [13], Scanorama [12], Seurat v4 RPCA [30], Harmony [6], and fastMNN [10]). We also included the widely used Seurat v4 CCA method [30]. The methods were benchmarked across four real datasets (pancreas, lung atlas, human immune, and human/mouse immune) and two simulated ones. We used the same type of input (with and without HVG selection and with or without scaling) and output (batch-corrected gene expression matrix and/or embedding), datasets (*n* = 6) and performance metrics (*n* = 14) as in the original benchmark study [5]. In total, the scib-pipeline ran 210 tasks. Eleven of these failed: Coralysis given the HVG unscaled gene expression matrix as input for the simulation 1 dataset; Seurat v4 CCA given the full scaled gene expression matrix as input for the lung atlas and human/mouse immune datasets; and all of the Seurat v4 RPCA tasks for the human/mouse immune and simulation two datasets (Fig. 2A and B).

The trade-off between batch correction and bio-conservation was assessed by averaging the five batch correction metrics and the nine bio-conservation metrics for each set of input data and each integration method (Fig. 2A). Coralysis performed consistently well across the different datasets, demonstrating a competitive and balanced trade-off between batch correction and biological signal conservation (Fig. 2A–C and Supplementary Figs S2–S7). In general, the differences in performance observed between the datasets reflected the complexity of the integration tasks. The human pancreas data consisted of the same tissue (pancreatic islets) analysed using distinct single-cell library preparation methods, the lung atlas included transcriptionally similar but functionally different cell types from the lung airway and parenchyma (Supplementary Fig. S8A and B), and the human and cross-species immune datasets represented more complex integration challenges with multiple batches, tissues (blood and bone marrow) (Supplementary Fig. S8C), and even organisms (human and mouse) (Supplementary Fig. S8D). Among the two simulation scenarios, simulation 2 included more batches and fewer clusters with transcriptionally similar identities than simulation 1 (Supplementary Fig. S9A and B). Thus, it is not surprising that the benchmarked methods generally performed better in simulation 1 than in simulation 2 (Fig. 2A,B and Supplementary Fig. S6,S7).

Another important aspect of Coralysis is the small variation in its performance with respect to the input provided, compared with the other methods (Fig. 2D). Notably, Coralysis avoided the integration of similar yet distinct cell types (Supplementary Figs S10 and S11). For example, in the human immune data, Coralysis was able to separate CD14+ from CD16+ monocytes and NK from NKT cells (Supplementary Fig. S10C). Coralysis’s higher bio-conservation metrics, such as cell-type average silhouette width (ASW) and isolated labels (F1 and silhouette) (Fig. 2B), supported these observations. Cell-type ASW measures the density and separation of cell types, while the isolated label metrics assess the ability to integrate cell types shared by only a few batches. This highlights the potential of Coralysis to outperform current best-performing integration methods in tasks requiring the integration of subtle biological variation and/or cell types that are unevenly distributed across batches.

### Coralysis outperforms the other methods in integrating imbalanced cell types

**Figure 3.**
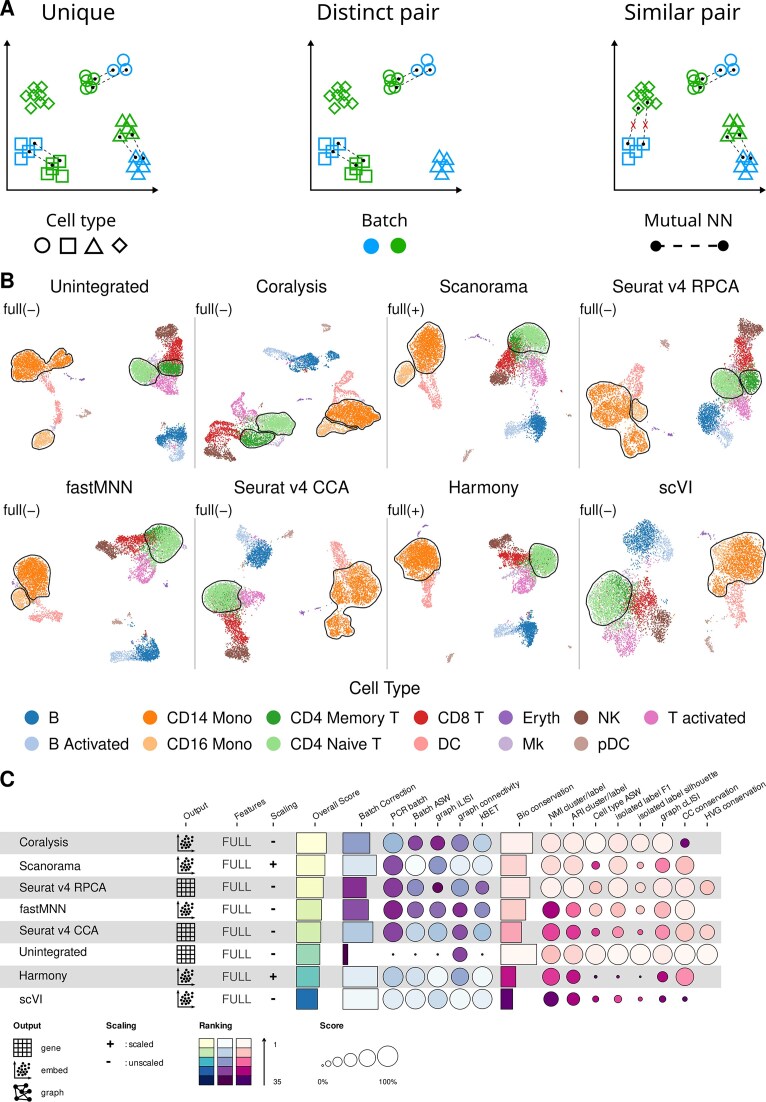
Coralysis outperforms best-performing methods for an integration task with batches with unshared similar cell-type pairs. (**A**) Representation of mutual nearest neighbours search on the low-dimensional space for integration scenarios in which batches do not share all cell types: unique (one cell type is unique to one of the batches), distinct pair (one distinct pair of cell types is unshared across batches, e.g. monocytes versus CD4 T cells), and similar pair (one similar pair of cell types is unshared across batches, e.g. CD4 naive versus CD4 memory). (**B**) UMAP projections highlighting the cell-type identity before and after integration of two PBMC scRNA-seq datasets through the scib-pipeline. (**C**) Performance ranking of integration methods by the overall score obtained with the scib-pipeline. Overall score corresponds to 0.4:0.6 weighted mean between batch-correction (blue/purple) and bio-conservation (pink) metrics, respectively. The two PBMC datasets consisted of one sample representing resting PBMCs (CTRL) and the other representing interferon-stimulated cells (STIM). CD4 naive T cells and CD14 monocytes were removed from the CTRL sample and CD4 memory T cells and CD16 monocytes were removed from the STIM sample. Unshared similar cell-type pairs are circumscribed by black lines. The best input-output combination was selected for every method. The label “full” represents input data with all features, and the minus and plus signs correspond to unscaled and scaled data, respectively.

To further investigate the hypothesis that Coralysis outperforms the best-performing integration methods in tasks involving uneven or unshared but transcriptionally similar cell types, we envisioned three possible scenarios for unshared cell types across batches (Fig. 3A): cell types unique to one of the batches, cell types unique to both batches that are transcriptionally distinct, and cell types unique to both batches that are transcriptionally similar. Since Scanorama, Seurat v4 CCA/RPCA, fastMNN, and Harmony perform integration in a joint low-dimensional space, they may overlook subtle biological differences that are not well preserved in reduced feature space, as opposed to probabilistic models such as Coralysis and scVI. Accordingly, although integration tasks involving the first two scenarios are unlikely to be wrongly integrated, it is likely that transcriptionally similar cell types will incorrectly correspond to mutual nearest neighbours.

We investigated this hypothesis using a dataset of two human PBMC samples: resting cells (CTRL) and interferon-stimulated cells (STIM) (Fig. 3B and Supplementary Fig. S12). Specifically, we considered two pairs of similar cell types CD4 naive and CD4 memory T cells, and CD14 and CD16 monocytes. In the CTRL sample, we retained CD16 monocytes and CD4 memory T cells but excluded CD14 monocytes and CD4 naive T cells. In the STIM sample, the opposite pattern of retention and exclusion was applied. Coralysis was the only method that could clearly separate CD14 from CD16 monocytes as well as naive from memory CD4 T cells (Fig. 3B and C, and Supplementary Figs S12 and S13). This observation is supported by both qualitative UMAP visualizations (Fig. 3B and Supplementary Fig. S12) and quantitative performance metrics based on bio-conservation and batch correction, in which Coralysis ranked first (Fig. 3C and Supplementary Fig. S13). Despite using a probabilistic model, scVI performed poorly, contrary to our expectations (Fig. 3B and C, and Supplementary Figs S12 and S13). This result supports our hypothesis, showing that Coralysis is a more suitable method for integrating unshared similar cell types.

Next, we sought to determine whether the superior performance of Coralysis in integrating unshared but similar cell types extended to scenarios involving imbalanced cell types. To this end, we used two batches of completely balanced PBMC datasets from Maan *et al.* [24], each comprising six cell types with exactly 200 cells per cell type per batch, to simulate cell type imbalance. B cells—a cell type that is transcriptionally distinct from the others—and CD14+ and FCGR3A+ monocytes—two transcriptionally similar cell types—were downsampled to 0% (complete absence), 5%, 10%, and 100% (fully balanced), and the resulting datasets were benchmarked using the scib-pipeline.

**Figure 4.**
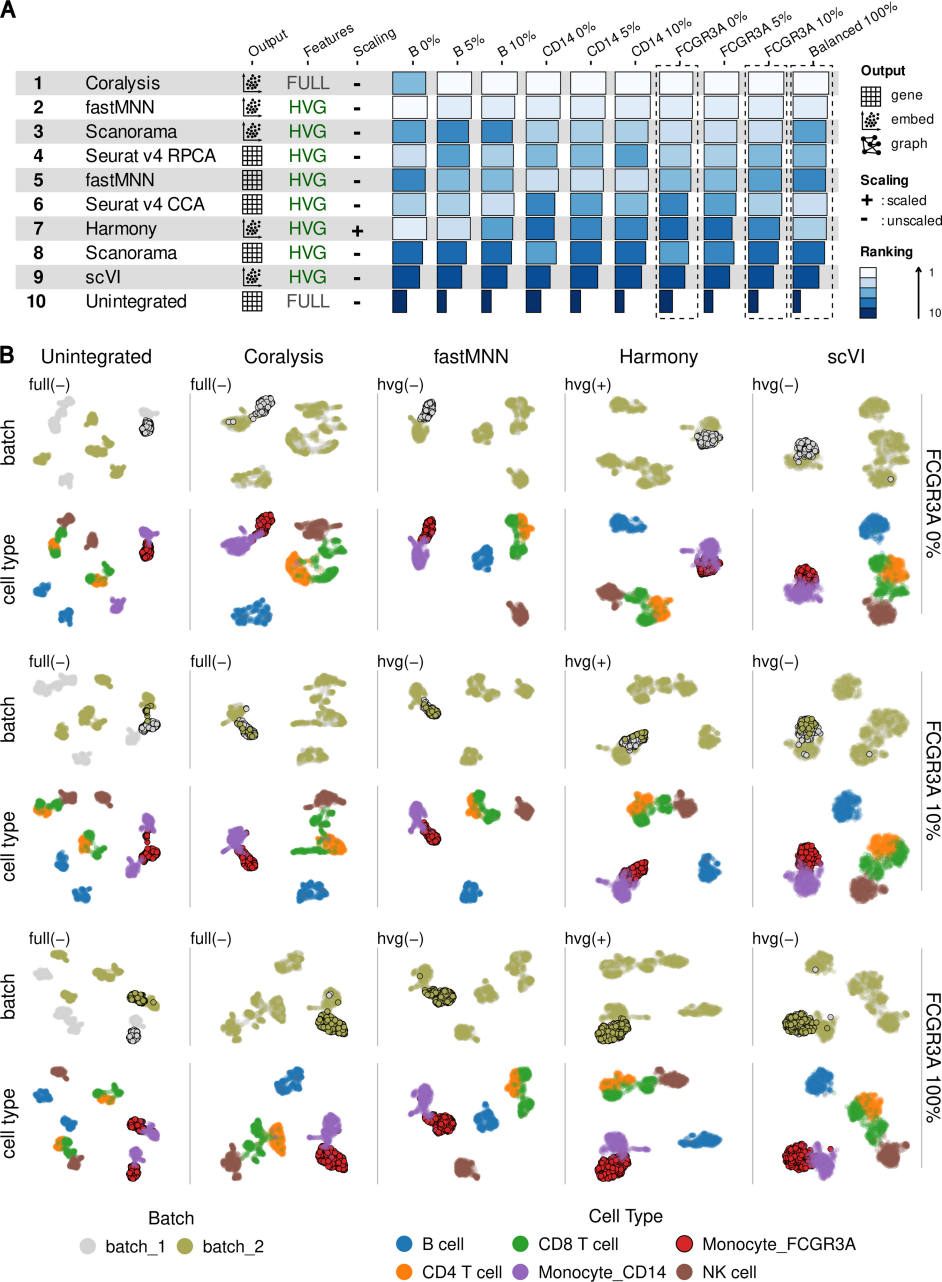
Coralysis outperforms best-performing methods for cell type imbalance integration tasks. (**A**) Overall method rankings across nine cell type imbalance tasks and one fully balanced integration task, as assessed using the scib-pipeline. The overall ranking represents the average rank across all ten tasks. The overall score is calculated as a weighted mean of batch correction and biological conservation metrics, with respective weights of 0.4 and 0.6. Each integration task involved the same two PBMC batch datasets, differing only in the specific cell type that was downsampled (B cells, CD14 monocytes, or FCGR3A monocytes). Downsampling was performed in batch “batch_2” to 0% (complete absence), 5%, or 10% of the target cell type. The “Balanced 100%” task refers to a control scenario with fully balanced PBMC batch datasets, containing 200 cells per cell type in each batch. (**B**) UMAP projections highlighting the batch (top) and cell-type (bottom) identity for the two top-performing and two bottom-performing methods, along the unintegrated projection, across the 0%, 10%, and 100% FCGR3A monocyte imbalance tasks (i.e. FCGR3A monocytes completely absent in “batch_2”, or downsampled to 10% in “batch_2”, and fully balanced across batches).

Coralysis outperformed all other integration methods across all tasks, except for the B cells 0% task, where it ranked fifth – still performing better than Scanorama and scVI (Fig. 4A). FastMNN ranked as the overall second-best method, showing good performance across all the datasets (Fig. 4A). In contrast, Harmony and scVI again performed poorly, particularly in tasks involving the integration of the transcriptionally similar monocyte populations (Fig. 4A and B). The advantage of Coralysis over FastMNN is especially evident in the integration of imbalanced FCGR3A + monocytes, where Coralysis clearly separates CD4 + and CD8 + T cells – unlike the other methods (Fig. 4B and Supplementary Fig. S14). Overall, this benchmark of cell type imbalance integration demonstrates that Coralysis is well-suited for integrating both unshared similar cell types and imbalanced cell types.

### Coralysis integrates heterogeneous single-cell proteomics datasets

**Figure 5.**
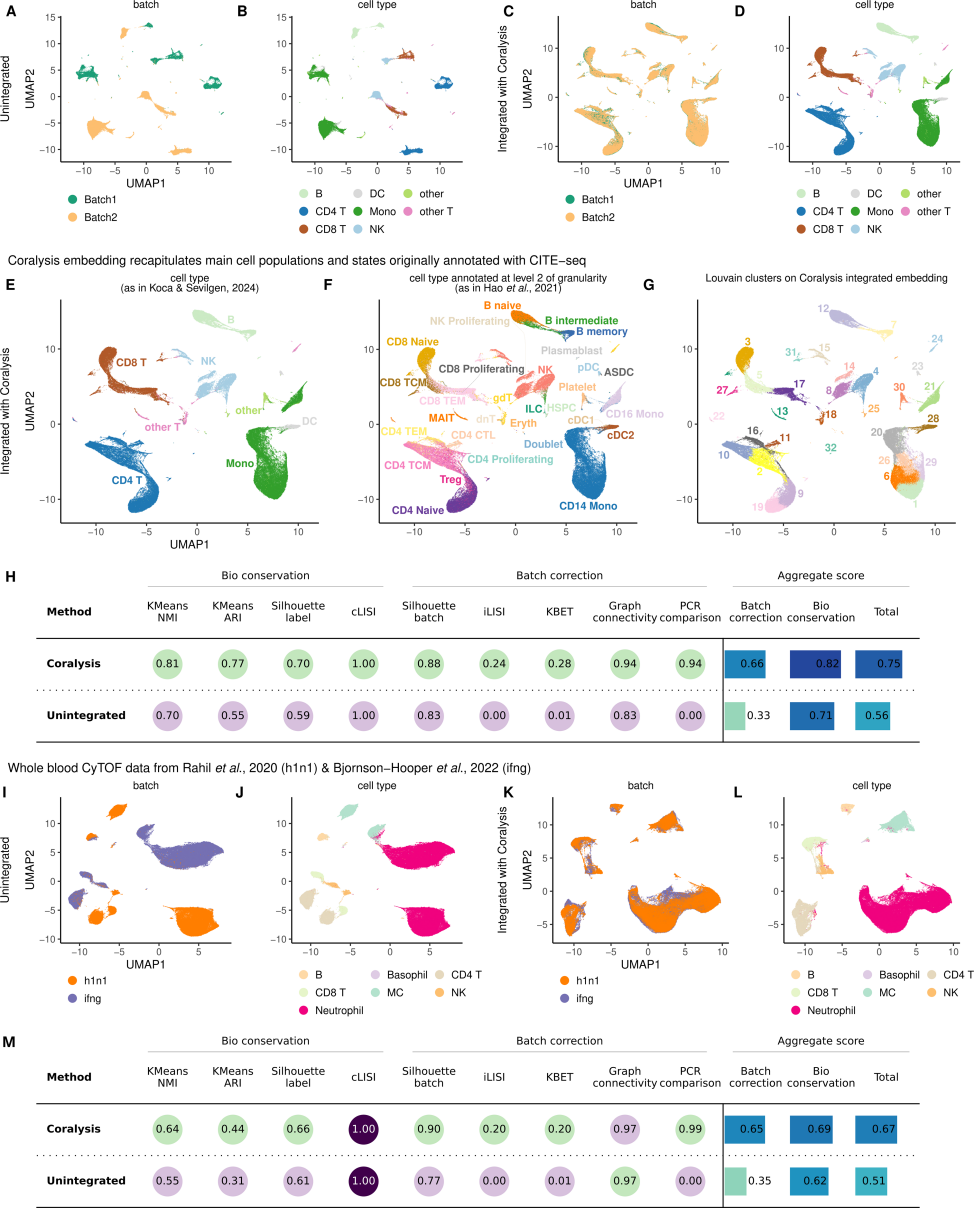
Coralysis enables the integration of heterogeneous single-cell proteomics datasets from different technologies. UMAP of unintegrated ADT data of human PBMCs originally published by Hao *et al.* [30] highlighting (**A**) the batch and (**B**) the cell-type label identities as given in Koca and Sevilgen [29]. UMAP of the same ADT data of human PBMCs after performing integration with Coralysis highlighting (**C**) the batch and (**D**) the cell-type label identities. Integrated UMAP with cells labelled as in (**E**) Koca and Sevilgen [29], as in (**F**) Hao *et al.* [30] at level 2 of granularity and with (**G**) the Louvain clusters computed on the Coralysis integrated embedding. (**H**) Assessment of integration performed with Coralysis on the ADT dataset provided by the scib-metrics Python package using as ground-truth the cell-type labels given in Koca and Sevilgen [29]. UMAP of unintegrated CyTOF data of whole blood from patients infected with influenza (*h1n1*) or cells stimulated with interferon-γ (*ifng*) originally published by Rahil *et al.* [31] and Bjornson-Hooper *et al.* [32], respectively, highlighting (**I**) the batch and (**J**) the cell-type label identities as given in Koca and Sevilgen [29]. UMAP of the same CyTOF data of human blood cells after performing integration with Coralysis highlighting (**K**) the batch and (**L**) the cell-type label identities. (**M**) Assessment of integration performed with Coralysis on the CyTOF dataset with the scib-metrics package.

Although Coralysis has been developed for the purpose of integrating and/or clustering single-cell transcriptomic datasets, it can also be applied to other single-cell modalities. To demonstrate this, we decided to apply Coralysis integration to single-cell proteomic data from two rapidly evolving technologies: ADTs and CyTOF. The ADT data were from human PBMC samples from eight HIV-infected donors (P1–8) reported by Hao *et al.* [30], collected at three timepoints to follow both vaccinated and unvaccinated individuals (228 proteins across 161 764 cells). The normalized data were integrated across two batches: the first comprising donors P1–4, and the second comprising donors P5–8 (Fig. 5A), as defined by Koca and Sevilgen [29]. Based on the eight higher-level cell-type labels available from the same study (Fig. 5B), Coralysis integrated all cell populations well (Fig. 5C and D). The high granularity observed independently of the batch (Fig. 5C and E) motivated us to look further into the more granular cell populations using the fine-grained cell-type annotations from the original study (at level 2 of granularity), which had been obtained using CITE-seq with both ADTs and RNA-seq data modalities [30] (Fig. 5E and F). Interestingly, several of the Louvain clusters identified with the Coralysis integrated embedding using only the ADT data matched the fine-grained cell populations identified with CITE-seq multi-modal data (Fig. 5F and G), for example, clusters 13 (MAIT), 21 (CD16 Mono), 23 (pDC), 18 plus 24 (gdT), and 30 (platelets) (Fig. 5F and G). Furthermore, despite the reduced feature space (228 proteins), Coralysis was able to preserve the differentiation trajectory, from naive to effector cell states, observed in B cells and CD4/CD8 T cells (Fig. 5F). These results were supported by the quantification of bio-conservation and batch correction using objective metrics, which showed a great improvement in batch correction without compromising bio-conservation for any of the ground-truth cell-type labels (Fig. 5H and Supplementary Fig. S15).

The CyTOF datasets were from human whole-blood samples reported by Rahil *et al.* [31] and Bjornson-Hooper *et al.* [32], including 35 donors infected with H1N1 influenza across 11 time points (*h1n1* dataset, 39 proteins across 102 147 cells), and samples from 86 healthy individuals stimulated with IFN$\gamma $ (*ifng* dataset, 39 proteins across 114 175 cells) (Fig. 5I and J). As shown in the integrated UMAP projections (Fig. 5K and L), Coralysis successfully integrated each immune cell type, including the less abundant basophils (0.5% of the cells—1075 cells), across the highly heterogeneous CyTOF datasets. These results were further supported by the improved scores for the bio-conservation and batch correction metrics when compared with the unintegrated baseline (Fig. 5M). Overall, these results demonstrated that Coralysis can accurately integrate single-cell proteomics data, even with a low number of features, such as in CyTOF.

### Coralysis achieves high reference-mapping accuracy under diverse query-reference scenarios

The ICP models generated by training a single-cell dataset with Coralysis can be used to predict the cluster identities of related, unannotated single-cell datasets, enabling reference-mapping. To understand the strengths and limitations of the Coralysis reference-mapping method, we assessed its accuracy across four query-reference scenarios: (i) imbalanced cell types, (ii) unshared cell types, (iii) unrepresented batches, and (iv) a varied strength of the batch effect.

To assess the performance of Coralysis under the first two scenarios of imbalanced and unshared cell types, two PBMC samples from Maan *et al.* [24] were used; the batch 1 sample of the data was used as the reference, and batch 2 as the query. Both samples consisted of six cell types (B cells, CD4 T cells, CD8 T cells, CD14 monocytes, FCGR3A monocytes, and NK cells), with each cell type containing the same number of cells (*n* = 200). For the scenario with imbalanced cell types, we iteratively down-sampled each cell type in the reference to 10% and predicted the cell-type labels by projecting against the reference after training with ICP (Supplementary Fig. S16A). The centroid cross-batch distance (Euclidean) in the PCA space between the reference and query cell types was quantified to highlight their differences across the cell types (Supplementary Fig. S16B). This is particularly important since the Coralysis reference-mapping method relies on the distances between query cell types projected onto the reference PCA to classify cell types. As expected, the closest query cell-type centroid to each reference cell-type centroid corresponded to the cell type matching the reference cell-type label (Supplementary Fig. S16B). The overall accuracy ranged between 85% and 96% (Supplementary Fig. S16C), being lowest for CD8 T cells and highest for B cells (Supplementary Fig. S16D and E). The accuracies were consistently lower for the down-sampled cell types, particularly for CD8 T cells (Supplementary Fig. S16C and Supplementary Fig. S17A–F). The CD8 T cells were mostly misclassified as CD4 T cells (Supplementary Fig. S16D), likely because of their transcriptional similarity, which was reflected in the smallest centroid cross-batch distance observed between the reference and query cell types (Supplementary Fig. S16B). Interestingly, the lowest Coralysis confidence scores for this classification task, corresponding to the proportion of *k* neighbours from the winning class, were associated with the CD8 T-cell misclassifications (Supplementary Fig. S16D).

In the scenario with unshared cell types, we conducted a similar analysis but, instead of downsampling, each reference cell type was iteratively ablated (Supplementary Fig. S18A and B). The classification accuracy dropped compared with that in the previous scenario, ranging between 80% and 82% (Supplementary Fig. S18C), primarily because of the absence of the ablated cell type in the reference. This led to misclassifications against the closest reference cell type (Supplementary Fig. S18B and Supplementary Fig. S19A–F). Overall accuracy was lowest when B cells were ablated and highest when CD14 monocytes were ablated (Supplementary Fig. S18D and E). In the absence of these cell types in the reference, B cells and CD14 monocytes were mostly classified against the closest reference cell-type centroids, CD4 T cells and FCGR3A monocytes (also known as CD16 monocytes), respectively (Supplementary Fig. S18B, D, and E).

**Figure 6.**
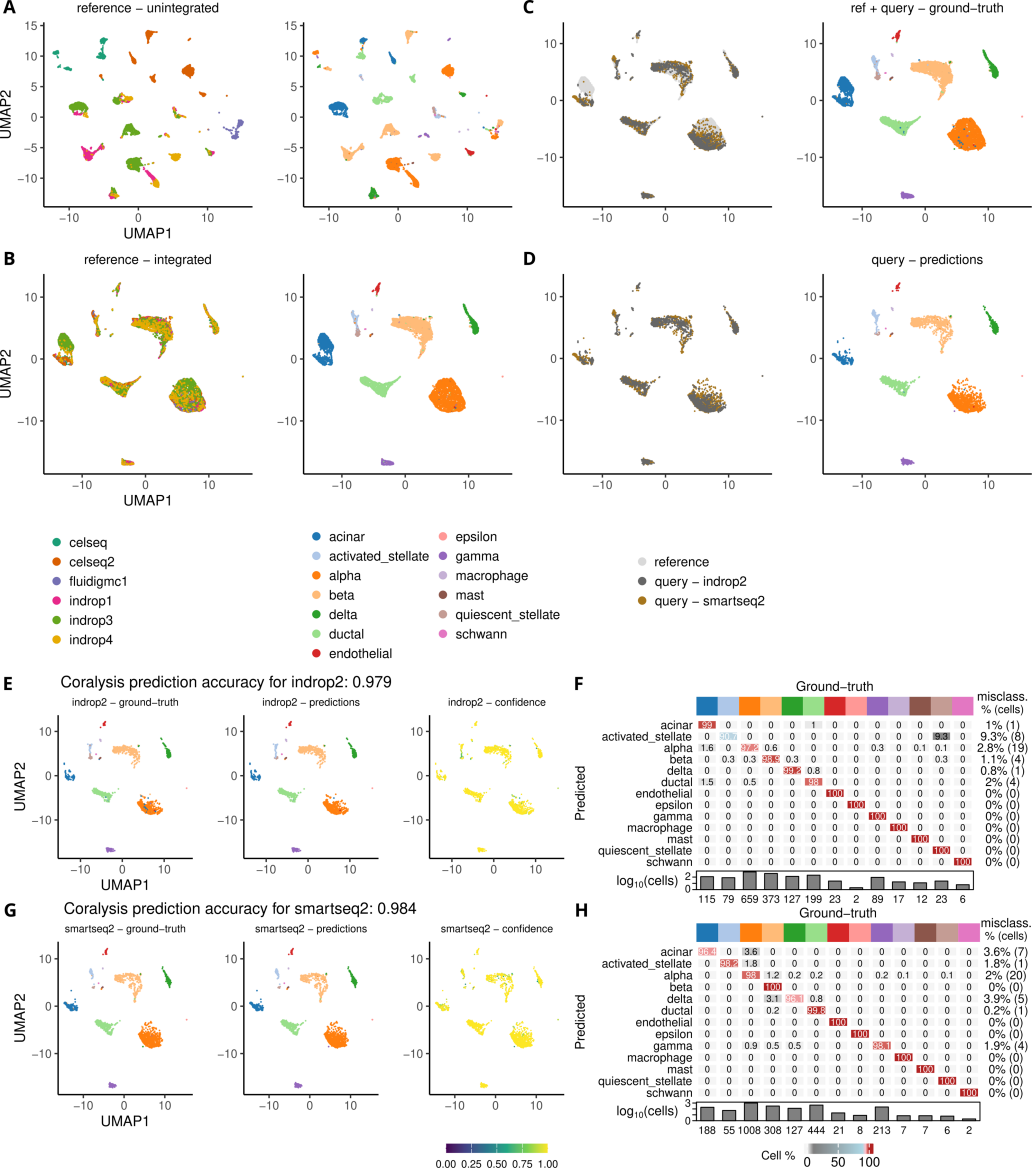
Accuracy of Coralysis reference-mapping method for a query-reference scenario of an unrepresented batch. (**A**) Unintegrated pancreatic reference UMAP highlighting the batch—sequencing libraries—and cell-type labels (left–right). (**B**) Integrated pancreatic reference UMAP obtained with Coralysis highlighting the respective batch and cell-type labels (left–right). (**C**) Queries projected onto the reference integrated UMAP with the Coralysis reference-mapping method. Colours highlight dataset identity (i.e. reference and query [indrop2 and smartseq2] labels [left], and ground-truth cell-type labels [right]). (**D**) Queries projected onto the reference UMAP with reference cells removed for clarity. Colours highlight query identity (dark gray for indrop2 and brown for smartseq2) and predicted cell labels (left–right). UMAP highlighting ground-truth, predictions, and confidence scores for (**E**) indrop2 and (**G**) smartseq2 queries projected onto the reference UMAP. Confusion matrix of predicted versus ground-truth cell-type labels for (**F**) indrop2 and (**H**) smartseq2 query predictions. The top colour bar represents cell-type labels. The confidence scores represent the proportion of *k* neighbours from the winning class (*k* = 10). The values in the confusion matrices correspond to the percentage of cells matching each other, while the values at the end of each row correspond to the number of total misclassifications in percentage and absolute number in parenthesis for every predicted cell type. The heatmap colours in the matrices represent the frequency of predicted cell types as a percentage. The barplots show the total number of cells for each cell type on a logarithmic scale (base 10) with the numbers indicated as labels.

In the unrepresented batch scenario, we built a reference of pancreatic islets by integrating six pancreatic scRNA-seq datasets from several technologies with Coralysis (Fig. 6A and B). The queries consisted of two pancreatic scRNA-seq datasets: indrop2 (1724 cells), which was represented in the reference, and smartseq2 (2394 cells), which was absent from the reference (Fig. 6C and D). The prediction accuracy was very high ($ \sim $98%), regardless of batch representation in the reference (Fig. 6E–H). Similarly, the confidence scores were high (Fig. 6E and G).

Finally, we tested the impact of varying strengths of batch effect on the reference-mapping accuracy of Coralysis. The same resting and interferon-stimulated PBMC samples from Kang *et al.* [28] as described in Section “Coralysis outperforms the other methods in integrating imbalanced cell types” were chosen to represent this scenario. The reference consisted of resting cells (6548 cells), while the query consisted of interferon-stimulated PBMCs (7451 cells). A PCA comprising the cells from both samples was performed to highlight the transcriptomic variation between cell types (Supplementary Fig. S20A). The variation observed along Principal Component 2 (PC2) was explained by the transcriptomic response to interferon stimulation, which was stronger for monocytes and DCs than for NK, T, and B cells (Supplementary Fig. S20A). To illustrate this more clearly, the distances between the reference and query cell type centroids were depicted in a heatmap, highlighting the cell-type-to-cell-type distances (Supplementary Fig. S20B). Monocytes and DCs were the most dissimilar cell types, showing a clear separation from the remaining cell types, which were more similar to each other (Supplementary Fig. S20B). The correspondence between ground-truth cell-type labels and Coralysis predictions was high, with overall accuracy close to 90%. The exception was a fraction of CD4 memory T cells that were wrongly predicted to be CD4 naive T cells (Supplementary Fig. S20C–F). Notably, the accuracy of classification for cell types that responded more strongly to interferon stimulation, such as monocytes and DCs (Supplementary Fig. S20A), remained high. Instead, the accuracy depended more on the similarity between the query and reference cell types (Supplementary Fig. S20B), with CD4 memory T cells being most difficult to predict because of their transcriptional similarity to CD4 naive T cells (Supplementary Fig. S20E and F). The low confidence scores in the overlapping neighbourhoods highlighted these cell predictions that should be more carefully inspected (Supplementary Fig. S20F). In summary, Coralysis accurately maps cells across distinct query-reference scenarios, as long as the reference faithfully represents the query.

### Impact of reference dataset choice on Coralysis reference-mapping accuracy

In addition to diverse query-reference scenarios, the choice of dataset used as the reference can also impact the accuracy of reference-mapping. To assess this, we tested the effect of switching the reference and query datasets on Coralysis accuracy using the exact same query-reference scenarios described in the previous section.

Switching the reference from a pancreatic scRNA-seq dataset generated with indrop2 (Supplementary Fig. S21A–D) to smartseq2 (Supplementary Fig. S21E–H) had minimal impact on accuracy, with results ranging from ∼95% to 97%, respectively. However, changing the reference from resting (CTRL; Supplementary Fig. S21I–L) to interferon-stimulated (STIM; Supplementary Fig. S21M–P) PBMCs resulted in an accuracy drop of ~5%. This observation led us to investigate whether the drop was due to stochastic variability in independent Coralysis runs or intrinsic differences between the reference datasets. To this end, we performed a replicability experiment: each cell type in each dataset was randomly downsampled to 70% across ten independent runs, and Coralysis reference-mapping was applied (with default parameters) using either CTRL or STIM as reference (with 2000 HVGs), and the other as query, and vice versa. The median accuracy difference remained statistically significant at ~6%, indicating that the resting PBMCs are a more representative and stable choice for reference (Supplementary Fig. S22A). Furthermore, using CTRL as the reference led to lower variability in accuracy across replicates. Increasing the number of highly variable genes to 6000 led to a marginal accuracy improvement for both datasets, but the median accuracy difference remained statistically significant (∼6%, Supplementary Fig. S22B). This persistent difference, even with a 3-fold increase in HVGs, suggested that the discrepancy may lie in the coefficients selected by ICP. To test this, we re-ran the replicability experiment using 2000 HVGs but replaced the default L1-regularization (which promotes sparsity) with L2-regularization (which retains all coefficients with small, non-zero weights). This led to an overall improvement in accuracy for both CTRL and STIM references, reduced variation in accuracy within each reference, and eliminated the statistically significant performance difference between them (Supplementary Fig. S22C).

Most misclassifications when using STIM as reference (Supplementary Fig. S21M–P) involved CD14 monocytes (Supplementary Fig. S22D). With CTRL as the reference, L1-regularization selected informative markers for CD14 monocytes—such as *S100A9, S100A8*, and *PLA2G7*—that were expressed in both CTRL and STIM datasets (Supplementary Fig. S22E). In contrast, STIM as the reference selected *CCL8* (*β* = 0.48), which is only expressed in stimulated CD14 monocytes (Supplementary Fig. S22F), likely reducing generalizability to the query.

Finally, we tested the effect of switching reference and query datasets under conditions of cell type ablation or imbalance. Using the two PBMC batch datasets described in the previous section, we down-sampled each completely balanced cell type in either batch (used as reference) to 0% (complete absence), 5%, 10%, 25%, 50%, or 100% (fully balanced) to simulate cell type ablation or imbalance. The Pearson correlation of accuracy between reference-switching experiments across all downsampled datasets was 0.93, indicating that cell type imbalance or absence has negligible effect on Coralysis accuracy when switching reference and query (Supplementary Fig. S21Q).

In summary, Coralysis reference-mapping is robust to switching between reference and query datasets, showing only small to negligible differences in accuracy—differences that can be further minimized by using L2- instead of L1-regularization.

### Coralysis identifies rare cell populations previously missed among PBMCs

**Figure 7.**
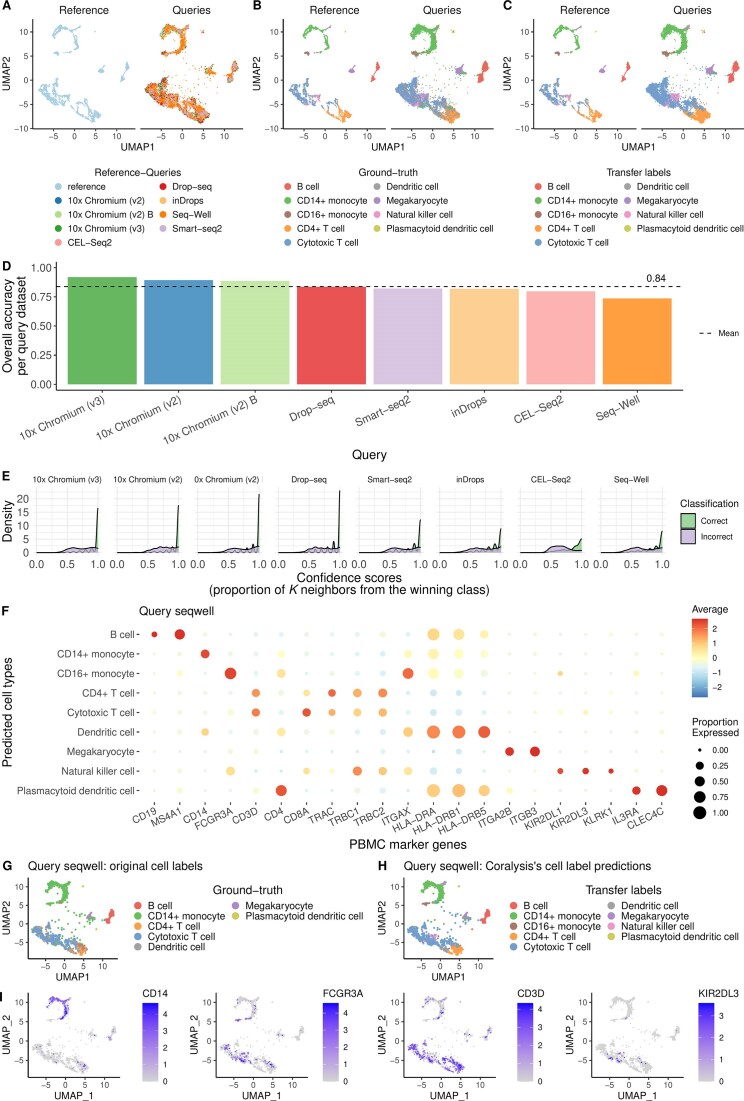
Coralysis identifies rare cell populations in a Seq-Well batch of PBMCs mapped against a 10x Chromium (v2) A reference PBMC scRNA-seq dataset from Ding *et al.* [34]. (**A**) Queries (right) projected onto the reference (left) UMAP. Queries consisted of eight scRNA-seq datasets of PBMCs sequenced with different technologies. The reference highlighted in light blue corresponds to PBMC scRNA-seq data prepared with 10x Chromium (v2) A technology. (**B**) Reference UMAP (left) and projected queries (right) highlighting the ground-truth cell-type labels as distributed through SeuratData software from Ding *et al.* [34]. (**C**) Reference UMAP (left) and projected queries (right) highlighting the cell type labels transferred from the reference to the queries with the Coralysis reference-mapping method. (**D**) Bar plot of overall accuracy per query dataset. The average accuracy across the eight queries is delimited with a dashed line. (**E**) Density plots of correct and incorrect classifications for each query dataset. (**F**) Dot plot showing the expression of marker genes for the predicted cell types of PBMCs for the query Seq-Well. Average expression was standardized by predicted cell type (row). Proportion expressed refers to the proportion of cells expressing the marker gene in the cell-type group. UMAP of the query Seq-Well projected onto the reference highlighting (**G**) the ground-truth cell-type labels and (**H**) the predicted cell labels by Coralysis. (**I**) UMAP of query Seq-Well showing the expression of marker genes of CD14+ monocytes (*CD14*) versus CD16+ monocytes (*FCGR3A*) as well as cytotoxic T cells (*CD3D*) versus natural killer cells (*KIR2DL3*) (from left to right).

Next, we more closely investigated the ability of Coralysis to identify fine-grained cell populations across heterogeneous scRNA-seq datasets. For this purpose, we selected nine PBMC datasets sequenced using different technologies [34]. As a reference, we selected one of the samples, which was sequenced using 10x Chromium (v2) A, whereas the remaining eight samples were used as queries (sequenced using 10x Chromium (v2), 10x Chromium (v2) B, 10x Chromium (v3), CEL-Seq2, Drop-seq, inDrops, Seq-Well, and Smart-seq2). The 27 753 query cells were successfully mapped onto the 3222 reference cells (Fig. 7A), with only minor disagreement between the ground-truth (Fig. 7B) and predicted cell labels (Fig. 7C). The mean accuracy per query dataset was 84% (Fig. 7D). Unsurprisingly, all of the 10x Chromium samples were above the mean as their data were obtained using the same technology as used for the reference. The accuracy decreased gradually from the droplet-based (Drop-seq and inDrops) to the plate-based datasets (Smart-seq2, CEL-Seq2, and Seq-Well) (Fig. 7D). The distribution of confidence scores for correct and incorrect classifications was similar across datasets. Correct classifications showed a high peak close to 1, whereas the incorrect classifications reached a smaller peak right after 0.5, generally followed by a uniform distribution towards 1 (Fig. 7E). These results support the use of confidence scores as a measure of classification reliability, particularly for scores below 0.75, for which the distribution of incorrect classifications exceeds that of correct ones.

We next investigated why the lowest accuracy was observed with the Seq-Well dataset. Encouragingly, all of the predicted cell types were supported by the expression of their respective marker genes (Fig. 7F). Therefore, we then investigated whether the low accuracy could be because of originally wrong classifications, as the Seq-Well dataset comprised only seven cell types, while nine cell types were predicted (Fig. 7G and H). Indeed, Coralysis identified two rare populations in the dataset—CD16+ monocytes and NK cells—that had previously been completely missed. The identification of CD16+ monocytes, which were previously misclassified as CD14+ monocytes, was supported by the expression of *FCGR3A* (also known as *CD16*) and the absence of *CD14*. Similarly, the appearance of NK cells was supported by the expression of *KIR2DL3* and the absence of *CD3D*, a marker of cytotoxic T cells (Fig. 7I). These results demonstrated the ability of Coralysis to identify previously missed rare cell populations.

### Coralysis cell cluster probabilities enable cell-state identification

Finally, we explored the applicability of the Coralysis cell cluster probabilities for inferring cell states and their differential expression programs. The Coralysis cell cluster probability corresponds to the likelihood that a cell belongs to its assigned cluster. This probability serves as a measure of confidence of the cluster assignment and as a proxy for similarity. Therefore, we hypothesized that highly distinct cells, representing terminally differentiated or steady states, would exhibit higher probabilities, while transient cells or intermediately differentiated ones would show lower probabilities, as they represent cells transitioning between steady states.

**Figure 8.**
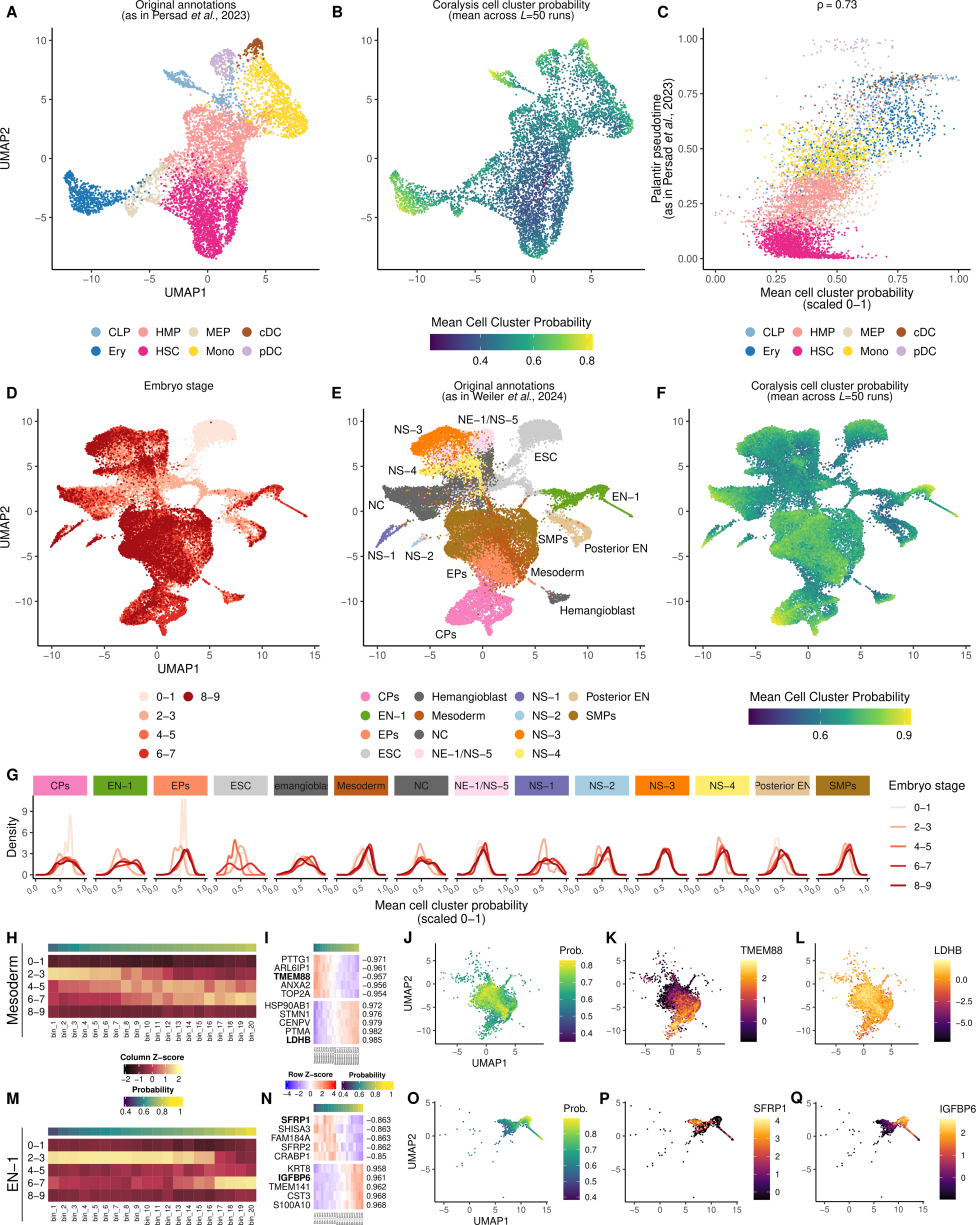
Coralysis cell cluster probability enables the identification of terminally differentiated cell types. UMAP projection of CD34+ bone marrow cells highlighting (**A**) cell-type labels as originally given by Persad *et al.* [36] (CLP: common lymphoid progenitor, HMP: haematopoietic multipotent progenitor, MEP: megakaryocyte-erythroid progenitor, cDC: conventional dendritic cells, Ery: erythroid, HSC: haematopoietic stem cells, Mono: monocytes, pDC: plasmacytoid dendritic cells) and (**B**) Coralysis cell cluster probability. Mean probability across the 50 independent ICP runs (i.e. *L* = 50). (**C**) Positive correlation between Coralysis cell cluster probability and Palantir pseudotime (*ρ* = 0.73) as provided in Persad *et al.* [36]. UMAP projection of embryoid body development integrated with Coralysis highlighting (**D**) the embryonic stage, (**E**) the cell-type labels as given originally by Weiler *et al.* [39] (CPs: cardiac precursors, EN: endoderm, EP: epicardial precursor, ESC: embryonic stem cell, haemangioblast, mesoderm, NC: neural crest, NE: neuroectoderm, NS: neuronal subtype, posterior EN: posterior endoderm, SMP: smooth muscle precursor), and (**F**) the Coralysis cell cluster probability. (**G**) Density plots of Coralysis cell cluster probability per embryonic stage for each cell type. Mean cell cluster probability was scaled to the range of 0–1. (**H**) Distribution of mesoderm cells across embryonic stages and Coralysis cell cluster probability bins (*n* = 20). Cell cluster probability was divided into a total of 20 bins highlighted at the top of the heatmap. Cell numbers were transformed by column *Z*-score. (**I**) Expression of the top five negatively and positively correlated genes across the Coralysis cell cluster probability bins for mesoderm cells. Pearson coefficient was calculated between the mean cell cluster probability and the mean gene expression across the 20 bins. Top colour bar corresponds to the mean cell cluster probability per bin. The averaged gene expression is represented by *Z*-scores (by row). UMAP representation of mesoderm cells showing (**J**) cell cluster probabilities and gene expression (scaled by *Z*-score) of (**K**) *TMEM88* and (**L**) *LDHB*. (**M**) Distribution of EN-1 cells across embryonic stages and Coralysis cell cluster probability bins (*n* = 20). (**N**) Expression of the top five negatively and positively correlated genes across the Coralysis cell cluster probability bins for EN-1 cells. UMAP representation of mesoderm cells showing (**O**) cell cluster probabilities and gene expression (scaled by *Z*-score) of (**P**) *SFRP1* and (**Q**) *IGFBP6*.

To test this hypothesis, we ran Coralysis on data from differentiating human bone marrow CD34+ cells from Persad *et al.* [36] (Fig. 8A). As expected, the Coralysis cell cluster probabilities were higher for terminally differentiated cells, such as erythroid, lymphoid, dendritic and monocyte cell lineages, and lower for transient, rapidly transitioning cells such as haematopoietic stem cells and haematopoietic multipotent progenitor cells (Fig. 8B). This observation was supported by the positive Pearson correlation (*ρ* = 0.73) between Coralysis cell cluster probability and Palantir pseudotime (Fig. 8C).

Next, we performed integration on 31 029 embryonic stem cells from a developing human embryoid body, generated by Moon *et al.* [38], using Coralysis to study the local distribution of cell cluster probabilities throughout the differentiation process (Fig. 8D and E; annotations used as given in Weiler *et al.* [39]). As expected, terminally differentiated cells such as neural crest, endoderm-1 (EN-1), cardiac precursors, neuronal subtype-1, and haemangioblast cells had higher cell cluster probabilities (Fig. 8F). Notably, Coralysis preserved inter-stage transcriptomic differences, as exemplified by embryonic stem cells and neural crest cells, despite integrating cells by embryonic stage (Fig. 8D and E). This further highlighted the ability of Coralysis to identify similar cell types across imbalanced datasets while preserving local biological differences. Interestingly, Coralysis cell cluster probabilities were able to discriminate within-cell-type differences across embryonic stages for several cell types, with cells progressing from low to high probabilities as they transitioned from early to late developmental stages (Fig. 8G).

Overall, these observations supported the applicability of Coralysis cell cluster probabilities for inferring cell states and their differential gene expression programs, which are expected to be stage-dependent, with early-stage cells being more immature and proliferative than mature cells at later stages. To demonstrate this, we divided the Coralysis cell cluster probabilities into 20 evenly sized bins separately for each cell type and calculated the Pearson coefficient between the average cell cluster probability and the average gene expression across the bins to identify stage-specific genes. Among the top five negatively (early) and positively (late) correlated genes per cell type were genes related to the cell cycle (e.g. *CDK1* and *CDKN3*), proliferation (e.g. *MALAT1* and *PTMA*), migration (e.g. *S100A10*), and differentiation (e.g. *CKB* and *ANXA2*/*5*) (see Supplementary Fig. S23). For instance, mesoderm and EN-1 cells showed a good correlation of early to late developmental stages with cell cluster probability bins, allowing better resolution over their differential gene expression programs (Fig. 8H and M). Among the most negatively correlated early genes for mesoderm cells, transmembrane protein 88 (*TMEM88*) is known to inhibit the Wnt/β-catenin signalling pathway in order to allow the commitment of pre-cardiac mesoderm cells into cardiomyocytes [40] (Fig. 8I–K). Notably, this was supported by the co-localization of *TMEM88*-expressing cells with epicardial and cardiac precursors (EPs and CPs, Fig. 8E). Additionally, glycolysis-related genes, such as *LDHB*, which was upregulated in late mesoderm cells, have been shown to exhibit differential expression across developmental stages in mesoderm during mouse gastrulation [41] (Fig. 8L). Finally, in agreement with our findings, Vianello and Lutolf [42] found *SFRP1* and *IGFBP5*, the latter functionally similar to *IGFBP6*, as being upregulated in early and late endoderm, respectively (Fig. 8N–Q). Overall, these results highlighted the ability of Coralysis to identify functionally relevant cell states and their differential expression programs.

## Discussion

In this paper, we present Coralysis, a method for sensitive integration, reference-mapping, and cell-state identification across single-cell datasets through multi-level integration. It relies on an adapted version of our previously introduced ICP algorithm [15] to identify shared cell clusters across heterogeneous datasets via multiple rounds of divisive clustering. Similar to assembling a puzzle, multi-level integration enables ICP to blend the batch effects and separate cell types by focusing on low- to high-level features (see Fig. 1 and Supplementary Fig. S1). The trained ICP models can then be used for various purposes, including prediction of cluster identities of related, unannotated single-cell datasets through reference-mapping, and inference of cell states and their differential expression programs using the cell cluster probabilities that represent the likelihood of each cell belonging to each cluster. Coralysis performed consistently well across a wide range of integration tasks compared with current best-performing integration methods. It prioritizes bio-conservation over batch correction, outperforming existing methods in imbalanced integration tasks, particularly those involving datasets that do not share similar cell types. Furthermore, Coralysis exhibited minimal variation in performance irrespective of the input data type, partly because of its inherent feature extraction procedure, which employs L1-regularized logistic regression [15].

The feature extraction procedure of Coralysis is particularly relevant for the detection of diseased or perturbed cell states and rare populations, as feature selection methods might overlook features required to discriminate them. For example, the commonly used Seurat highly variable gene selection method relies heavily on low-expressed genes with high coefficients of variation representing technical noise that obscures subtle cellular variation [43]. Furthermore, Coralysis allows the retrieval of gene coefficients from ICP models for a given ICP run or clustering label using a majority voting approach. This confers the advantage of identifying cluster marker genes independently from differential expression analysis, which avoids the bias towards highly expressed genes representing false positives [44, 45]. Notably, logistic regression, which is at the core of ICP, was reported to be one of the best methods for identifying cluster markers among 59 recently benchmarked tools [46].

As the complexity of cell atlases increases in terms of the tissues, timepoints and physiological conditions represented, subtle biological differences between cells can become difficult to dissociate from batch effects, likely increasing the demand for such integration functionalities. The application of Coralysis is not limited to single-cell transcriptomics; it can also be applied to integrate data from other single-cell assays, such as ADTs or CyTOF, within the exciting and rapidly evolving field of single-cell proteomics (Fig. 5). The reduced feature space of these assays, typically ranging from dozens to a few hundred of features, did not prevent Coralysis from identifying fine-grained cell populations and preserving differentiation trajectories, nor from detecting rare populations, such as basophils (0.5%), in whole-blood CyTOF data. Coralysis’s ability to identify cell populations at high resolution is expected to be of great value as unbiased measurements of single-cell proteomes become more common with advances in mass spectrometry [47]. In the future, we aim to extend the application of the Coralysis integration method to other single-cell assays, such as ATAC-seq, and even across different modalities with shared features by testing different regularizations (e.g. Ridge instead of LASSO) and data input transformations.

The use of Coralysis also avoids laborious *de novo* integration when a reference trained with Coralysis is available. Reference-mapping is becoming routine as projects such as the Human Cell Atlas [48] and the Tabula Muris [49] work to decipher the identities of millions of cells. Robust methods are needed to accurately map, for instance, diseased cells against healthy references, perturbed cells, or cells generated by new single-cell technologies. Here, we demonstrated that Coralysis performs well across several query-reference scenarios, including cell-type imbalance, batch representation, and varied batch strength. We also showed that Coralysis is applicable to the identification of previously missed rare cell populations across query-reference datasets originating from different sequencing technologies. Additionally, the confidence scores help users to identify which cell-type predictions should be validated. Although Coralysis currently has limitations in detecting cells not represented in the reference, this is unlikely to present a major obstacle as references become increasingly comprehensive. Future developments should address this limitation, such as by comparing the similarity between query and reference model coefficients.

A long-standing challenge in single-cell biology is how to define cell type versus cell state and how to identify and distinguish them. Cell states correspond to discrete modes that a given cell type can assume under different stimuli [50]. As such, they share a common gene expression program, which places them under the same cell-type “umbrella” definition. For example, the CD8+ T-cell type can adopt the naive, memory or effector CD8+ T-cell state. Coralysis cell cluster probability aids in the identification of terminally differentiated and transient cell types. Similarly to “metacells”, cell cluster probability bins aggregate highly similar cells, representing primarily technical noise, and capture existing biological variation within cell types across bins. This can help identify cell states and their differential expression programs, such as those involved in early to late embryoid cell development or healthy versus diseased cells. We anticipate that Coralysis will extend and refine the catalogue of cell heterogeneity by improving the integration of imbalanced cell types and states, enabling a more faithful representation of the cellular landscape in complex single-cell experiments.

## Supplementary Material

gkaf1128_Supplemental_Files

## Data Availability

All datasets analysed in this study have been previously published and made publicly available by the original authors. Links to each dataset are provided in the methods section alongside their descriptions. All code used in this manuscript is publicly available in our GitHub repository: https://github.com/elolab. The specific resources include: Coralysis R package: https://github.com/elolab/Coralysis and https://doi.org/10.5281/zenodo.17248332. Coralysis Bioconductor package: https://www.bioconductor.org/packages/release/bioc/html/Coralysis.html. Benchmark forked from theislab/scib-pipeline: https://github.com/elolab/scib-pipeline and https://doi.org/10.5281/zenodo.17248681. R and Python scripts for reproducing analyses and figures: https://github.com/elolab/Coralysis-reproducibility and https://doi.org/10.5281/zenodo.17248532. Additionally, the Coralysis website is available at: https://elolab.github.io/Coralysis.
