# Supplementary material for: Coralysis enables sensitive identification of imbalanced cell types and states in single-cell data via multi-level integration
Source: Nucleic Acids Res. 2025 Nov 13;53(21):gkaf1128. doi: 10.1093/nar/gkaf1128 (PMC12614221; doi:10.1093/nar/gkaf1128)
Supplement: gkaf1128_Supplemental_Files [file gkaf1128_supplemental_files.zip › ms-supp-figs-coralysis.pdf]

Coralysis enables sensitive identification of imbalanced cell types and states in single-cell data via multi-level integration

António G.G. Sousa[*1,2,✉], Johannes Smolander[†1,2], Sini Junttila[‡1,2], and Laura L. Elo[§1,2,3,✉]

[1]Turku Bioscience Centre, University of Turku and Åbo Akademi University, Turku, Finland

[2]InFLAMES Research Flagship Centre, University of Turku, Turku, Finland

[3]Institute of Biomedicine, University of Turku, Turku, Finland

[✉]To whom correspondence should be addressed: António G.G. Sousa (aggode@utu.fi) & Laura L. Elo (laura.elo@utu.fi)

[*]aggode@utu.fi

[†]johannes.smolander@helsinki.fi

[‡]simaju@utu.fi

[§]laura.elo@utu.fi

Supplementary Figures

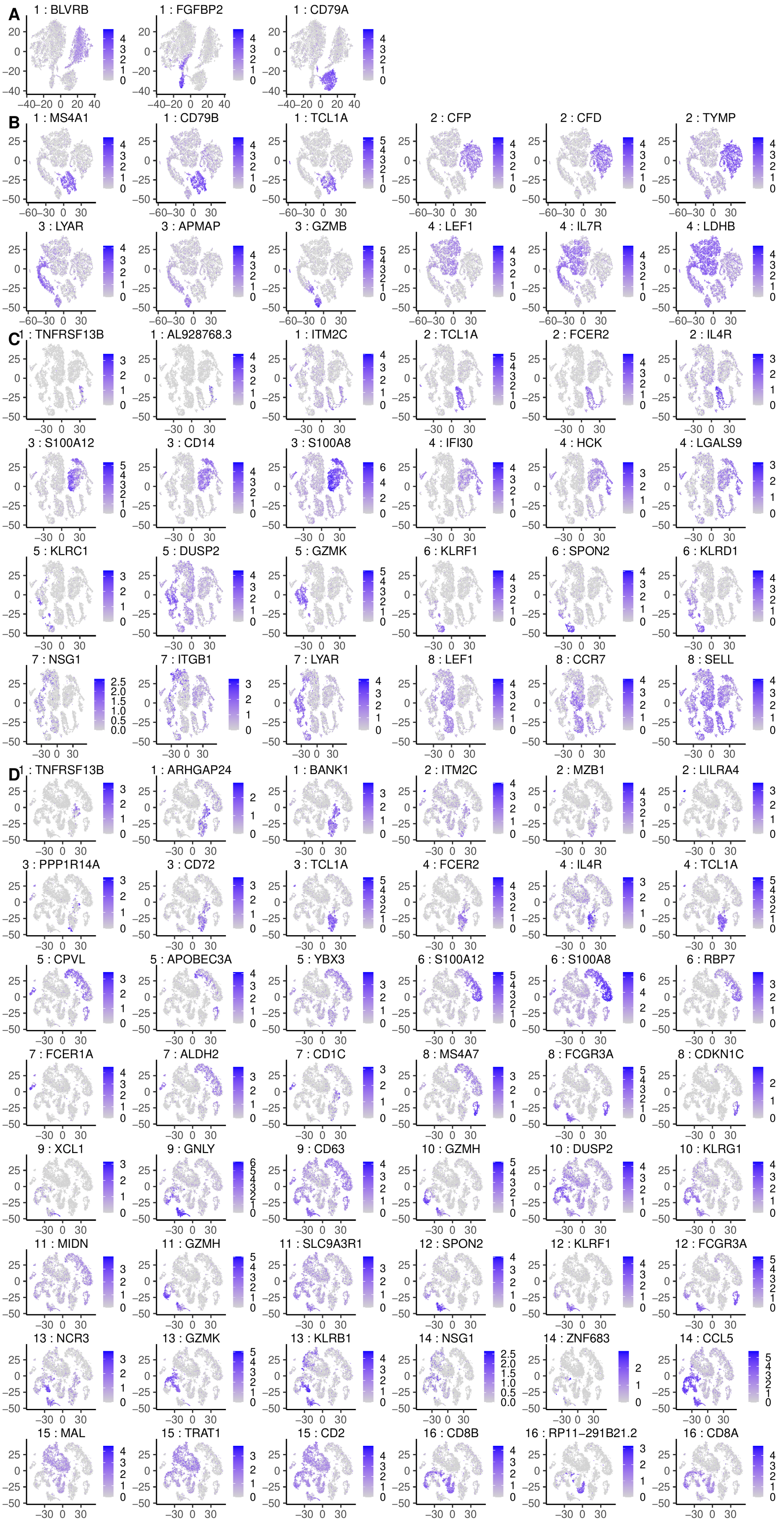
A
1 : BLVRB
1 : FGFBP2
1 : CD79A
B
1 : MS4A1
1 : CD79B
1 : TCL1A
2 : CFP
2 : CFD
2 : TYMP
3 : LYAR
3 : APMAP
3 : GZMB
4 : LEF1
4 : IL7R
4 : LDHB
C
1 : TNFRSF13B
1 : AL928768.3
1 : ITM2C
2 : TCL1A
2 : FCER2
2 : IL4R
3 : S100A12
3 : CD14
3 : S100A8
4 : IFI30
4 : HCK
4 : LGALS9
5 : KLRC1
5 : DUSP2
5 : GZMK
6 : KLRF1
6 : SPON2
6 : KLRD1
7 : NSG1
7 : ITGB1
7 : LYAR
8 : LEF1
8 : CCR7
8 : SELL
D
1 : TNFRSF13B
1 : ARHGAP24
1 : BANK1
2 : ITM2C
2 : MZB1
2 : LILRA4
3 : PPP1R14A
3 : CD72
3 : TCL1A
4 : FCER2
4 : IL4R
4 : TCL1A
5 : CPVL
5 : APOBEC3A
5 : YBX3
6 : S100A12
6 : S100A8
6 : RBP7
7 : FCER1A
7 : ALDH2
7 : CD1C
8 : MS4A7
8 : FCGR3A
8 : CDKN1C
9 : XCL1
9 : GNLY
9 : CD63
10 : GZMH
10 : DUSP2
10 : KLRG1
11 : MIDN
11 : GZMH
11 : SLC9A3R1
12 : SPON2
12 : KLRF1
12 : FCGR3A
13 : NCR3
13 : GZMK
13 : KLRB1
14 : NSG1
14 : ZNF683
14 : CCL5
15 : MAL
15 : TRAT1
15 : CD2
16 : CD8B
16 : RP11−291B21.2
16 : CD8A

Supplementary Figure S1. Expression of the top three positive gene coefficients for the ICP model corresponding to run 2 projected onto *t*-SNE. (**A–D**) Top three positive coefficients per cluster per clustering round level *K2* (**A**), *K4* (**B**), *K8* (**C**) and *K16* (**D**). The plot title highlights the cluster number and the respective gene coefficient. For every clustering round level a *t*-SNE was built from the concatenation of all cluster probability tables (*L*=50 ICP runs) from the respective clustering round.

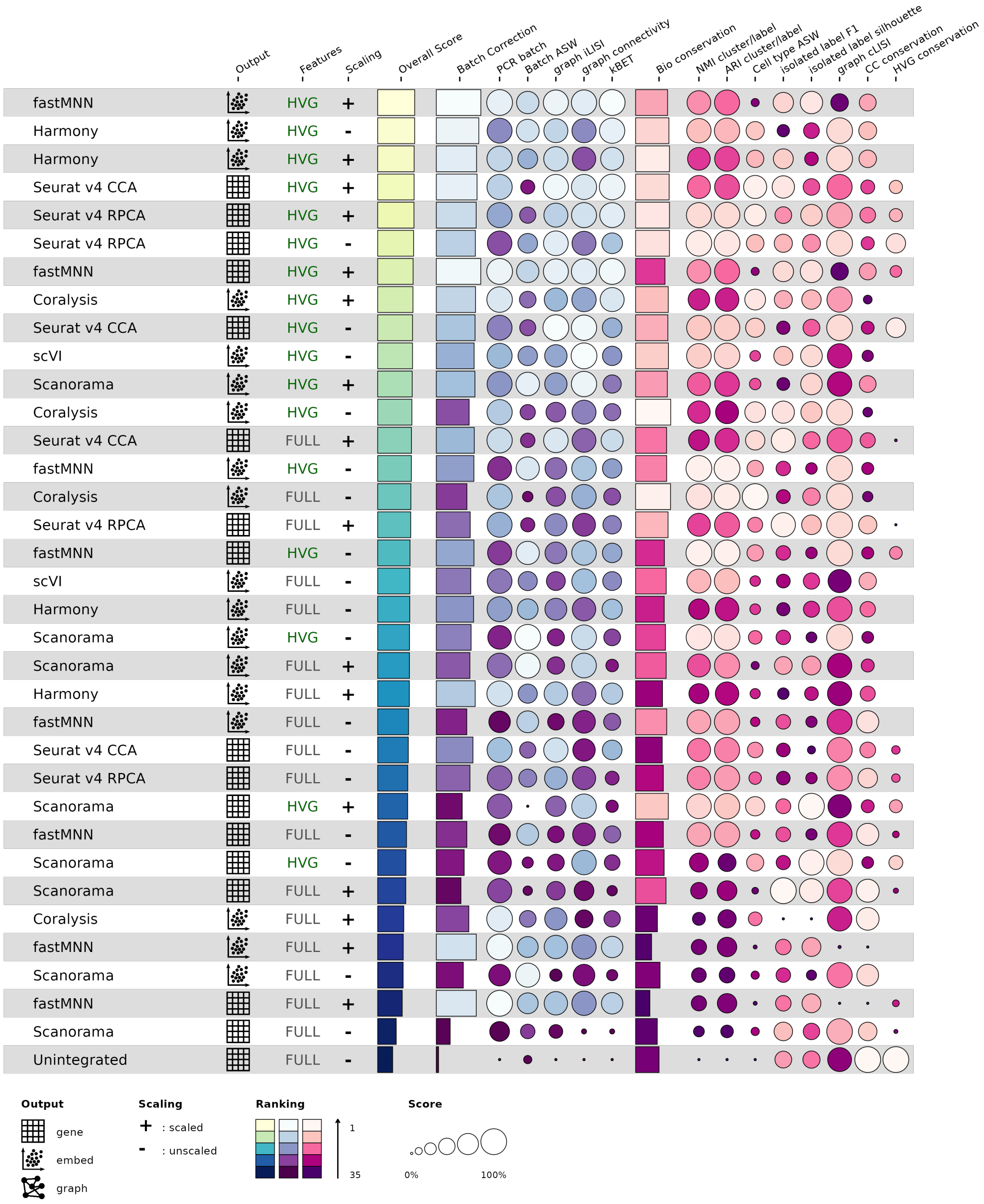

Supplementary Figure S2. Performance ranking of integration methods by the overall score obtained with the scib-pipeline for the pancreas dataset. Overall score corresponds to 0.4:0.6 weighted mean between batch-correction (blue/purple) and bio-conservation (pink) metrics, respectively.

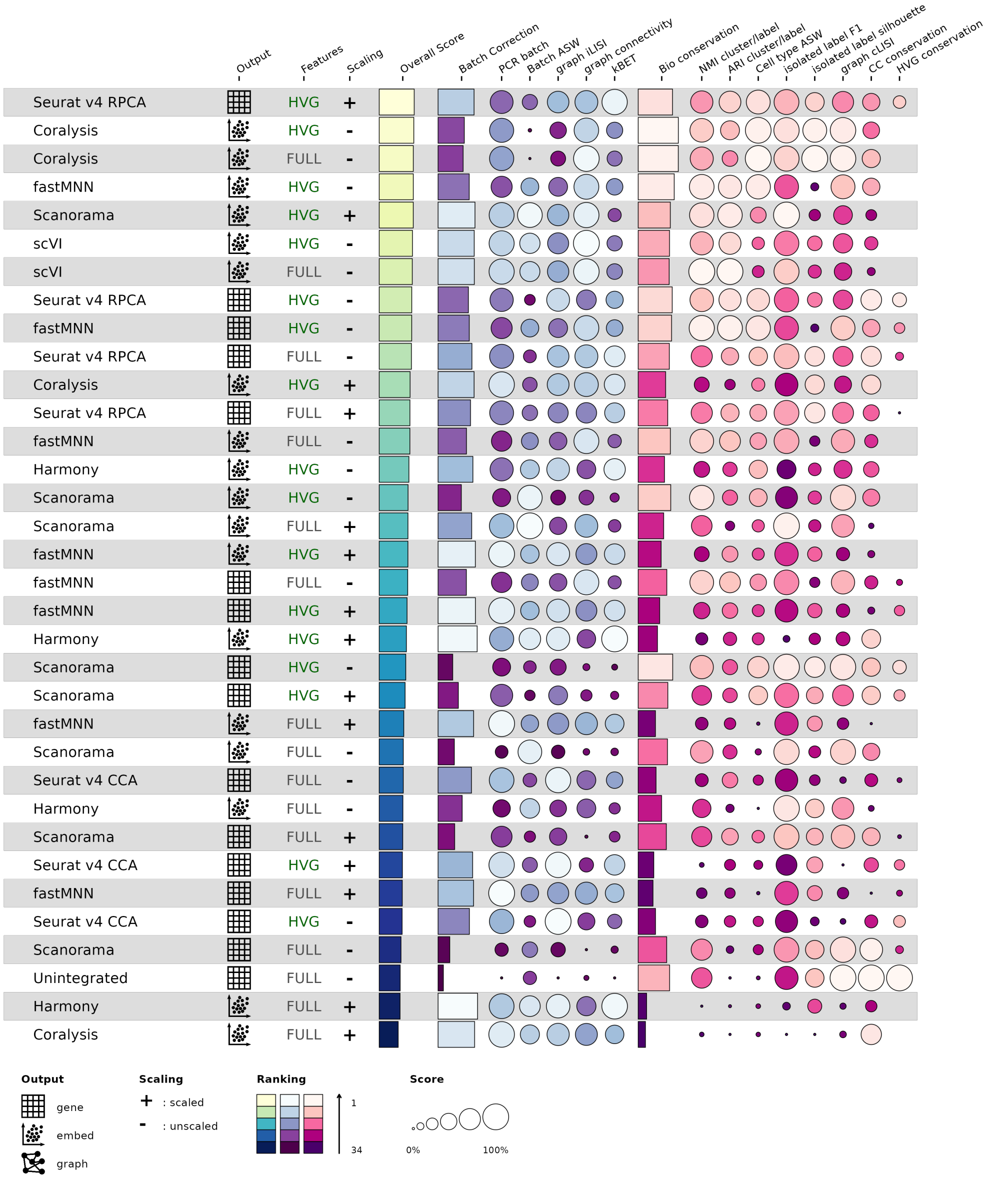

Supplementary Figure S3. Performance ranking of integration methods by the overall score obtained with the scib-pipeline for the lung atlas dataset. Overall score corresponds to 0.4:0.6 weighted mean between batch-correction (blue/purple) and bio-conservation (pink) metrics, respectively.

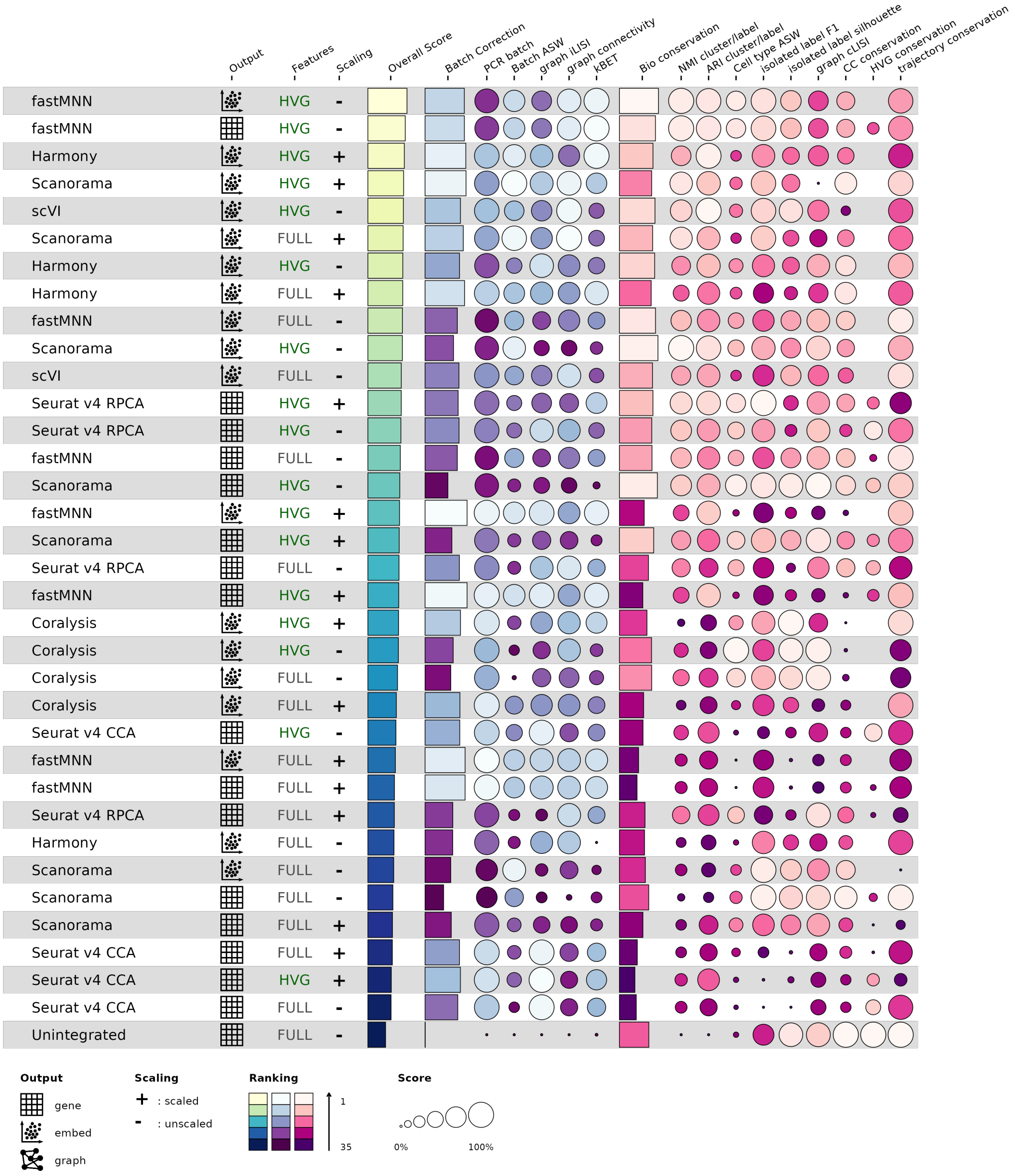

Supplementary Figure S4. Performance ranking of integration methods by the overall score obtained with the scib-pipeline for the human immune dataset. Overall score corresponds to 0.4:0.6 weighted mean between batch-correction (blue/purple) and bio-conservation (pink) metrics, respectively.

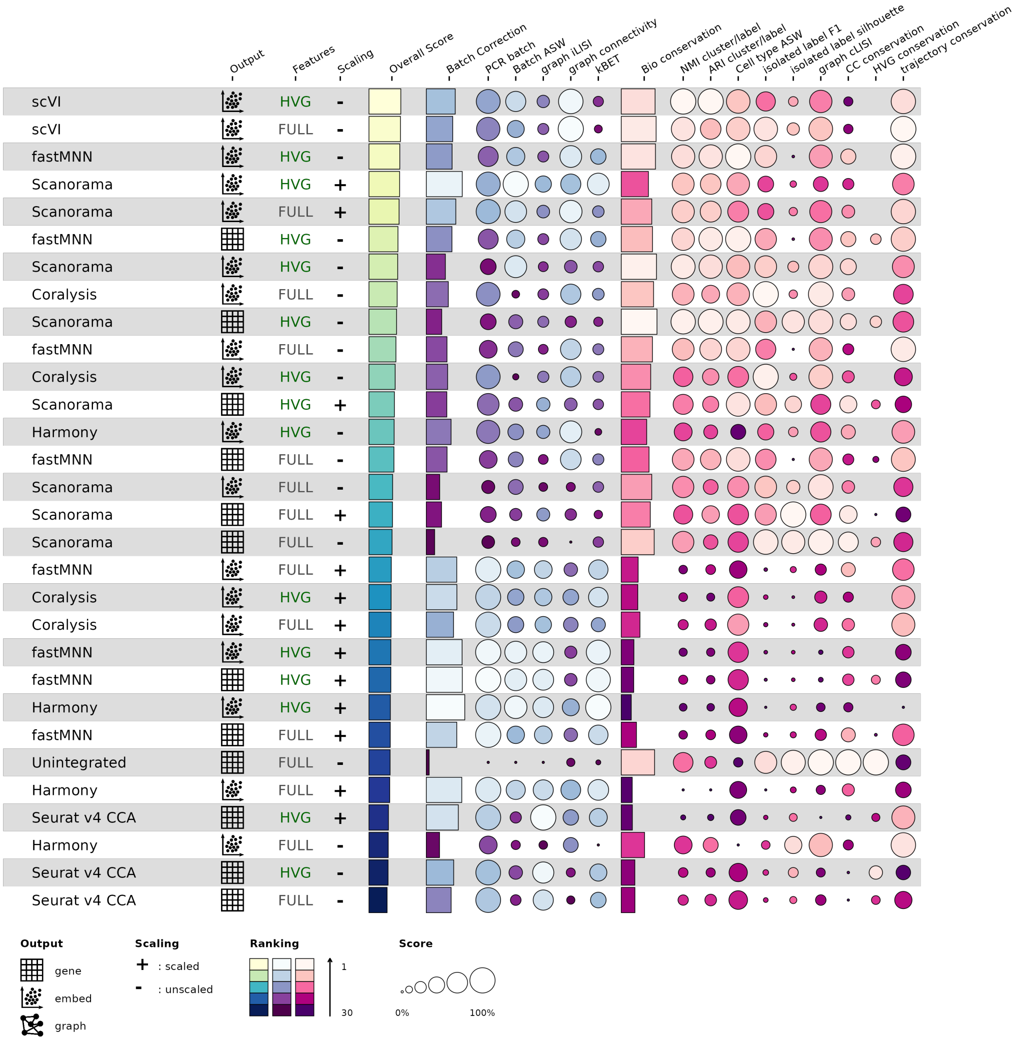

Supplementary Figure S5. Performance ranking of integration methods by the overall score obtained with the scib-pipeline for the human/mouse dataset. Overall score corresponds to 0.4:0.6 weighted mean between batch-correction (blue/purple) and bio-conservation (pink) metrics, respectively.

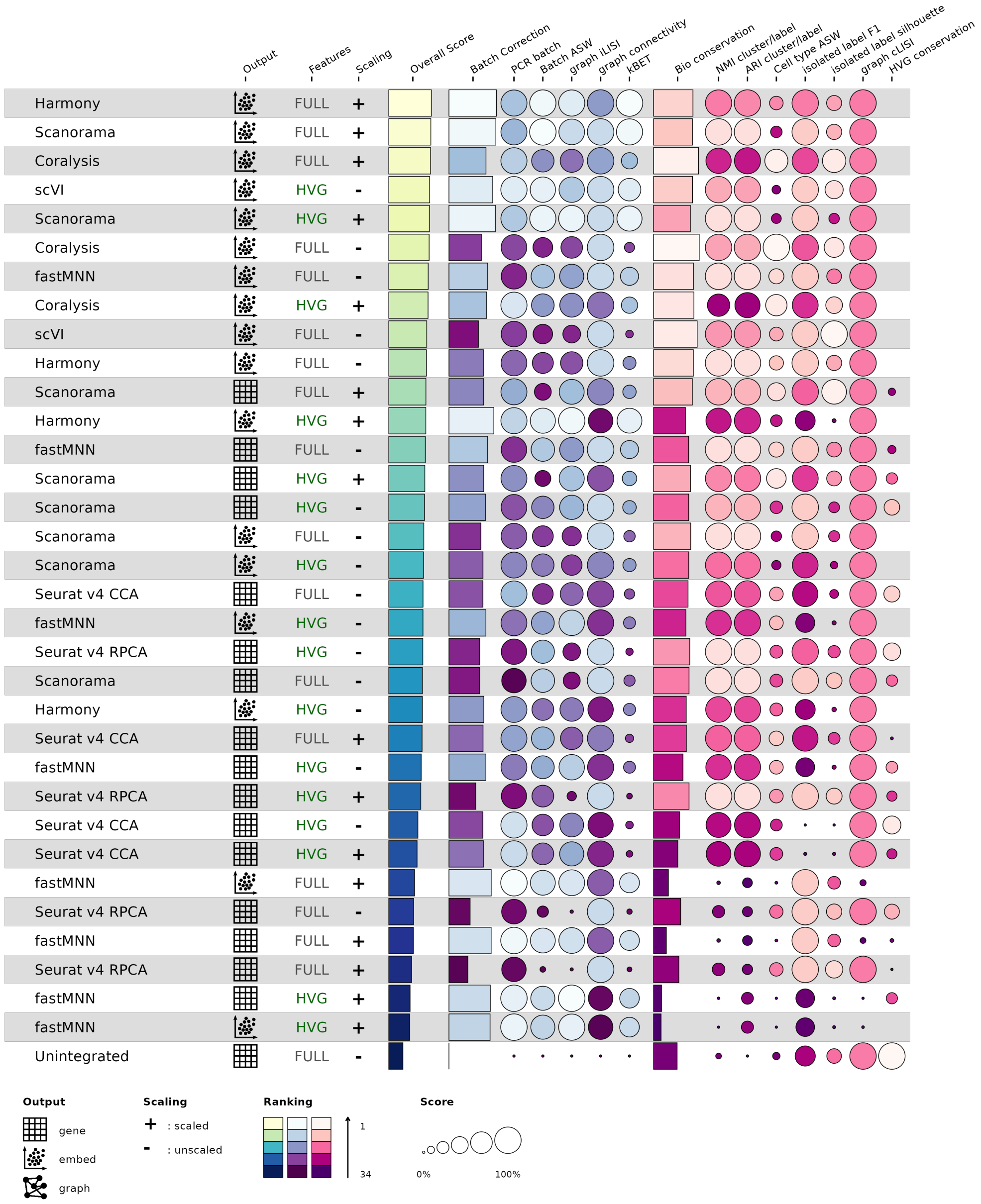

Supplementary Figure S6. Performance ranking of integration methods by the overall score obtained with the scib-pipeline for the simulation 1 dataset. Overall score corresponds to 0.4:0.6 weighted mean between batch-correction (blue/purple) and bio-conservation (pink) metrics, respectively.

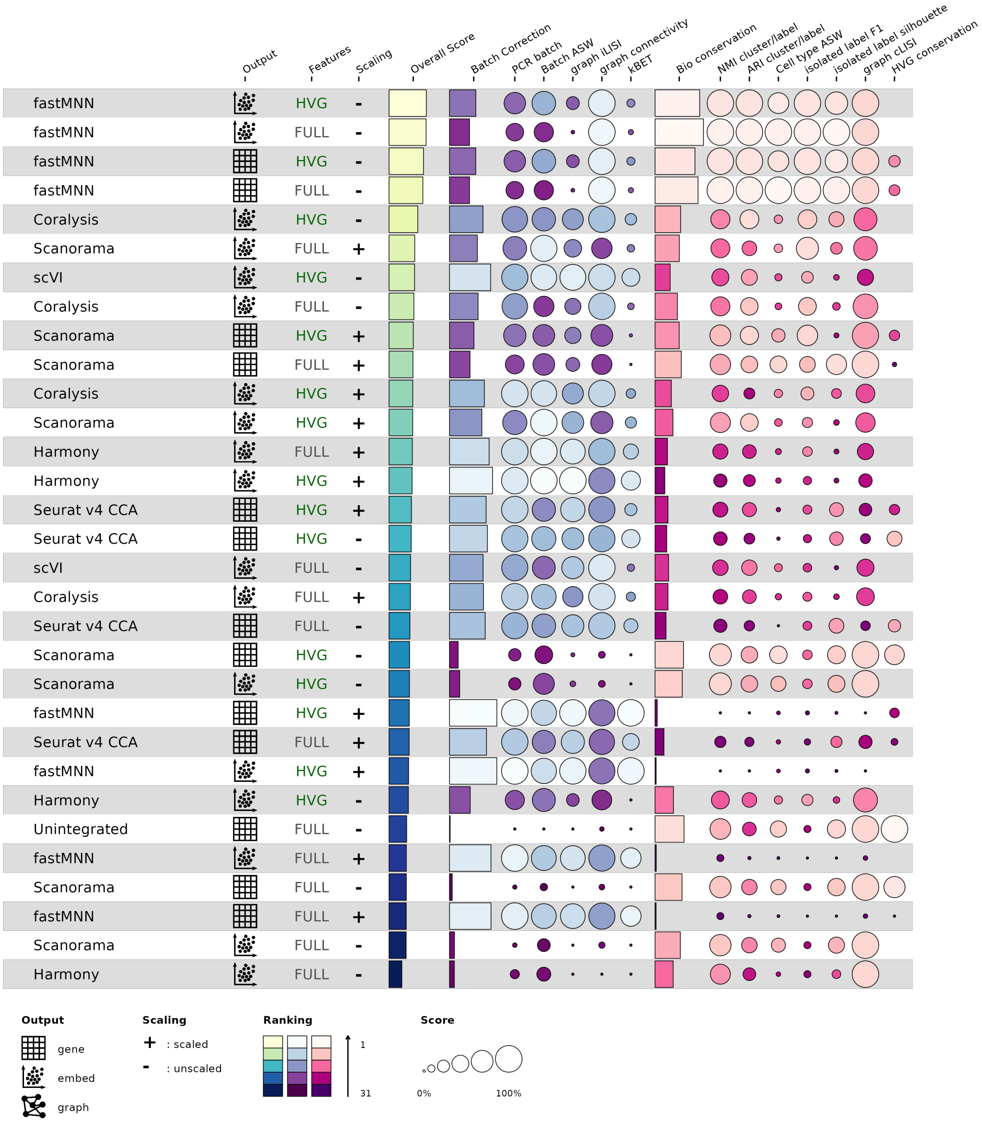

Supplementary Figure S7. Performance ranking of integration methods by the overall score obtained with the scib-pipeline for the simulation 2 dataset. Overall score corresponds to 0.4:0.6 weighted mean between batch-correction (blue/purple) and bio-conservation (pink) metrics, respectively.

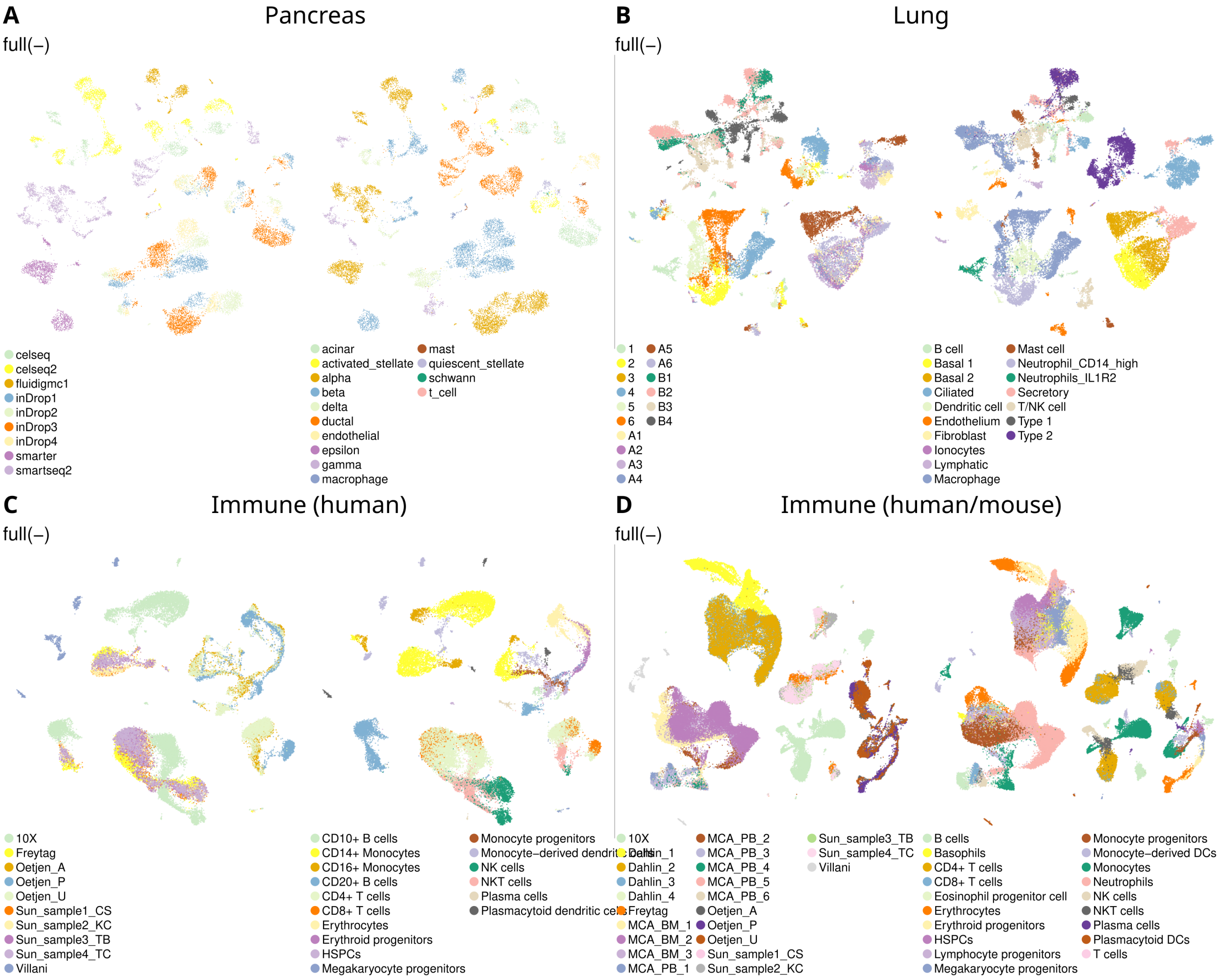

Supplementary Figure S8. Unintegrated UMAP projections for the four real datasets used to benchmark Coralysis obtained through the scib-pipeline. The datasets included were pancreas, lung atlas, human immune and human/mouse immune (**A-D**). The centered title corresponds to the dataset. The top left subtitle corresponds to the input data used, i.e., full unscaled gene expression matrix (full(-)). The left and right plots for each dataset highlight the batch and cell-type labels, respectively.

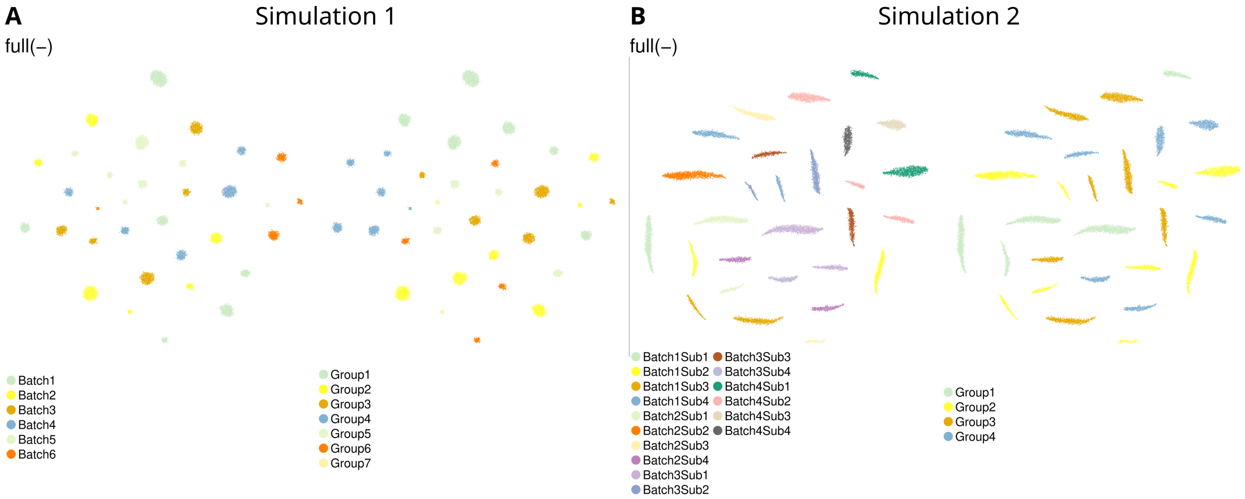

Supplementary Figure S9. Unintegrated UMAP projections for the two simulated datasets used to benchmark Coralysis obtained through the scib-pipeline. The datasets included were simulation 1 and 2 (**A-B**). The centered title corresponds to the data set. The top left subtitle corresponds to the input data used, i.e., full unscaled gene expression matrix (full(-)). The left and right plots for each dataset highlight the batch and cell-type labels, respectively.

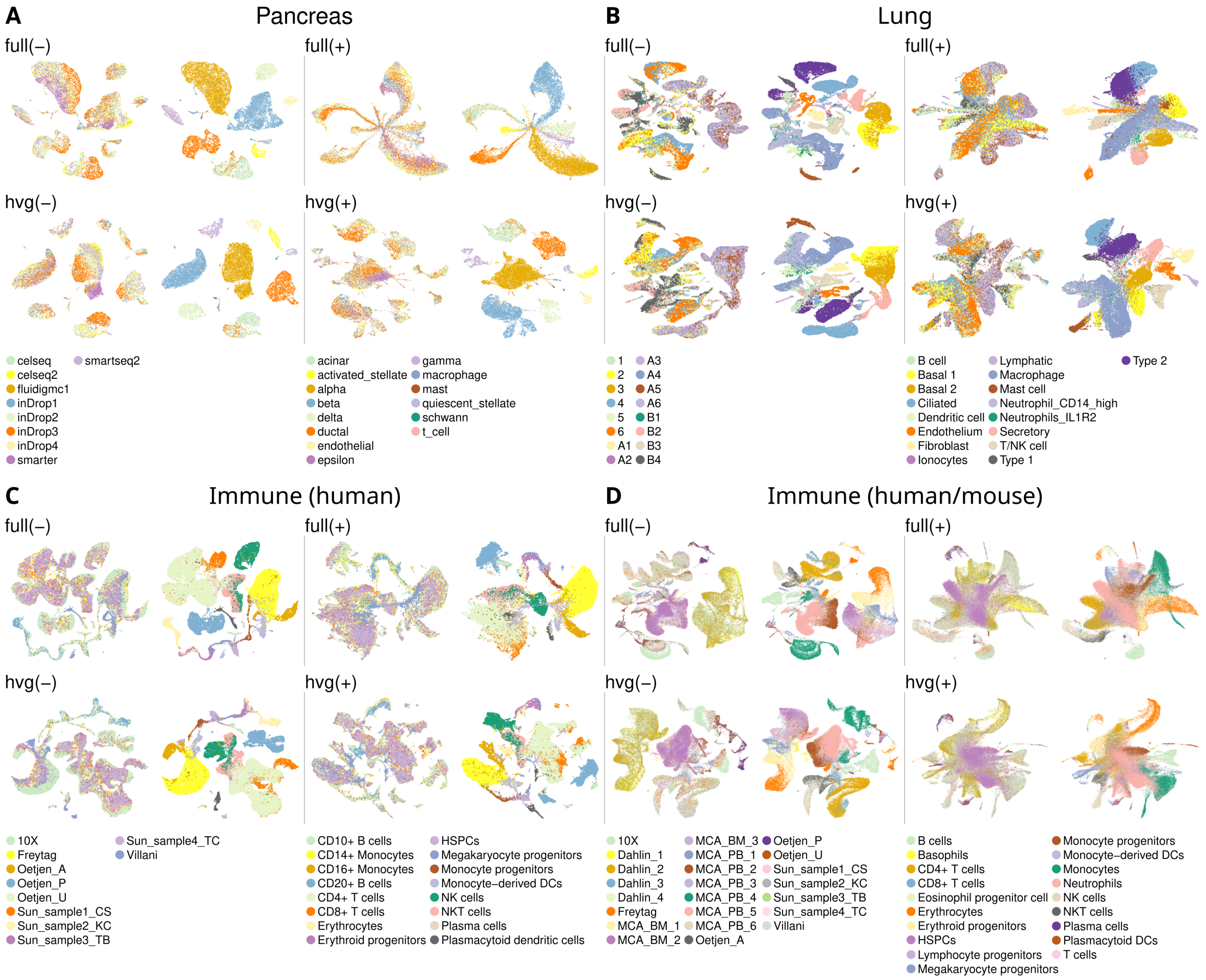

Supplementary Figure S10. Coralysis integrated UMAP projections for the four real datasets obtained through the scib-pipeline benchmark. The included datasets were pancreas, lung atlas, human immune and human/mouse immune (**A–D**). The top left subtitle corresponds to the input data used (from left to right, from top to bottom): full unscaled gene expression matrix (full(–)), full scaled gene expression matrix (full(+)), highly variable genes unscaled gene expression matrix (hvg(–)) and highly variable genes scaled gene expression matrix (hvg(+)). The left and right plots for each dataset for a given input data type highlight the batch and cell-type labels, respectively.

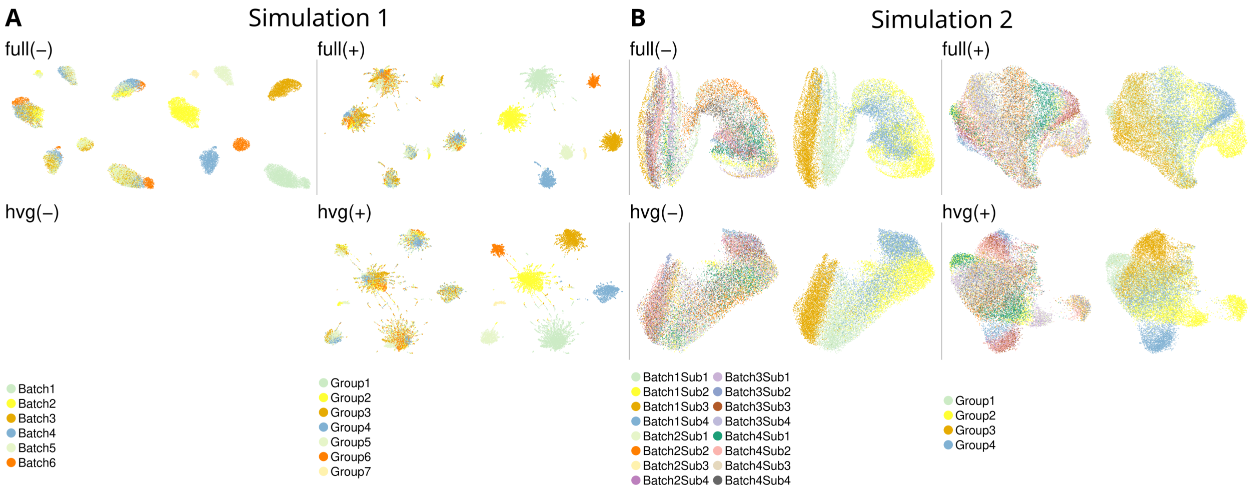

Supplementary Figure S11. Coralysis integrated UMAP projections for the two simulated datasets obtained through the scib-pipeline benchmark. The datasets included were simulations 1 and 2 (**A–B**). The top left subtitle corresponds to the input data used (from left to right, from top to bottom): full unscaled gene expression matrix (full(–)), full scaled gene expression matrix (full(+)), highly variable genes unscaled gene expression matrix (hvg(-)) and highly variable genes scaled gene expression matrix (hvg(+)). The left and right plots for each dataset for a given input data type highlight the batch and cell type labels, respectively. The integration task for the simulation 1 dataset, given the input hvg(–), failed.

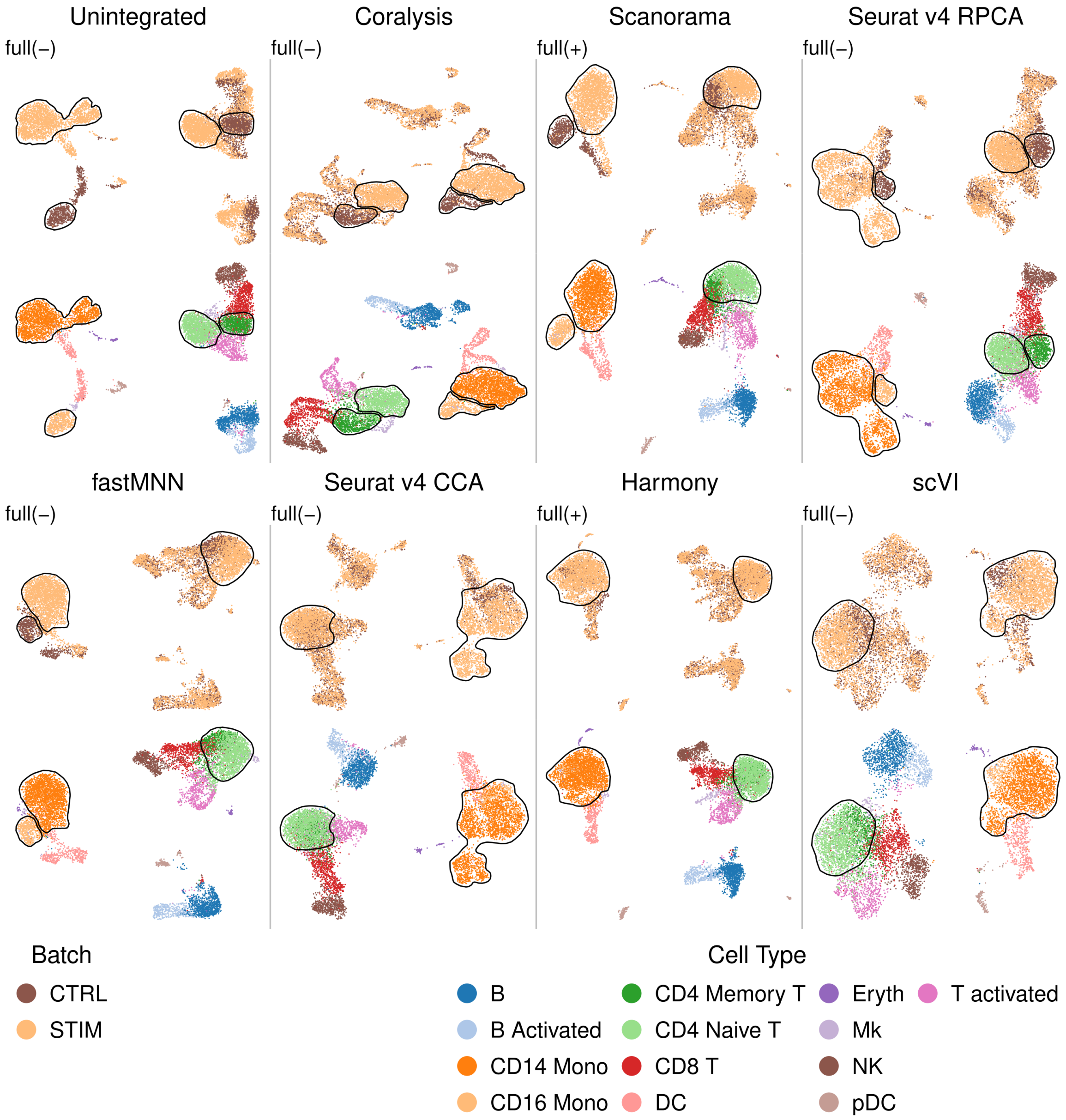

Supplementary Figure S12. UMAP projections highlighting the batch and cell-type identities before and after integration of two PBMC scRNA-seq datasets through the scib-pipeline. The two PBMC datasets consisted of one sample representing resting PBMCs (CTRL) and the other interferon-stimulated cells (STIM). CD4 naive T cells and CD14 monocytes were removed from the CTRL batch sample and CD4 memory T cells and CD16 monocytes from the STIM sample. Unshared similar cell type pairs are circumscribed by black lines. The best input-output combination was selected for every method. The label "full" represents input data with all features, and the minus and plus signs correspond to unscaled and scaled data, respectively.

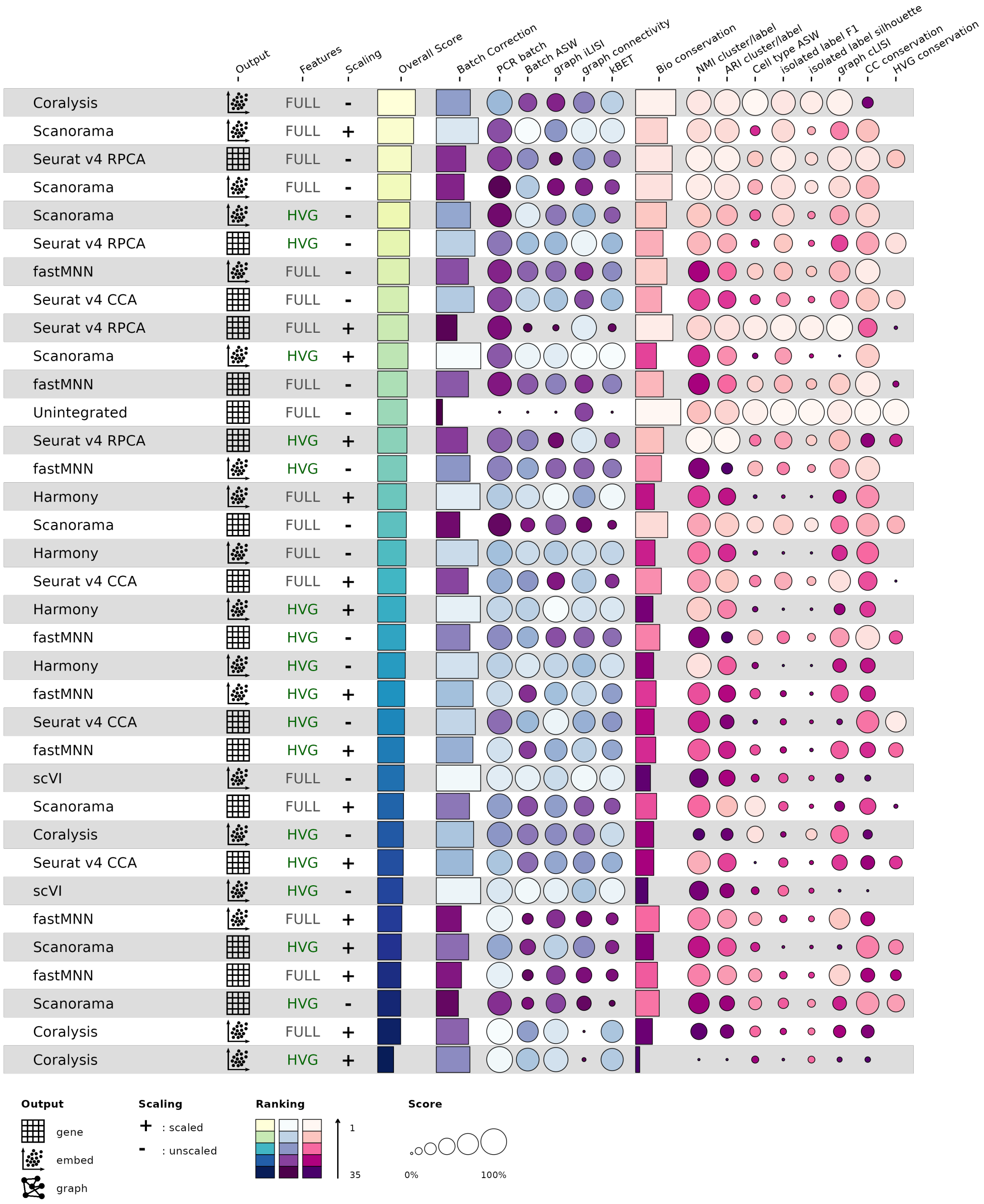

Supplementary Figure S13. Performance ranking of integration methods by the overall score obtained with the scib-pipeline for the two PBMCs scRNA-seq data sets with unshared similar cell type pairs. Overall score corresponds to 0.4:0.6 weighted mean between batch-correction (blue/purple) and bio-conservation (pink) metrics, respectively.

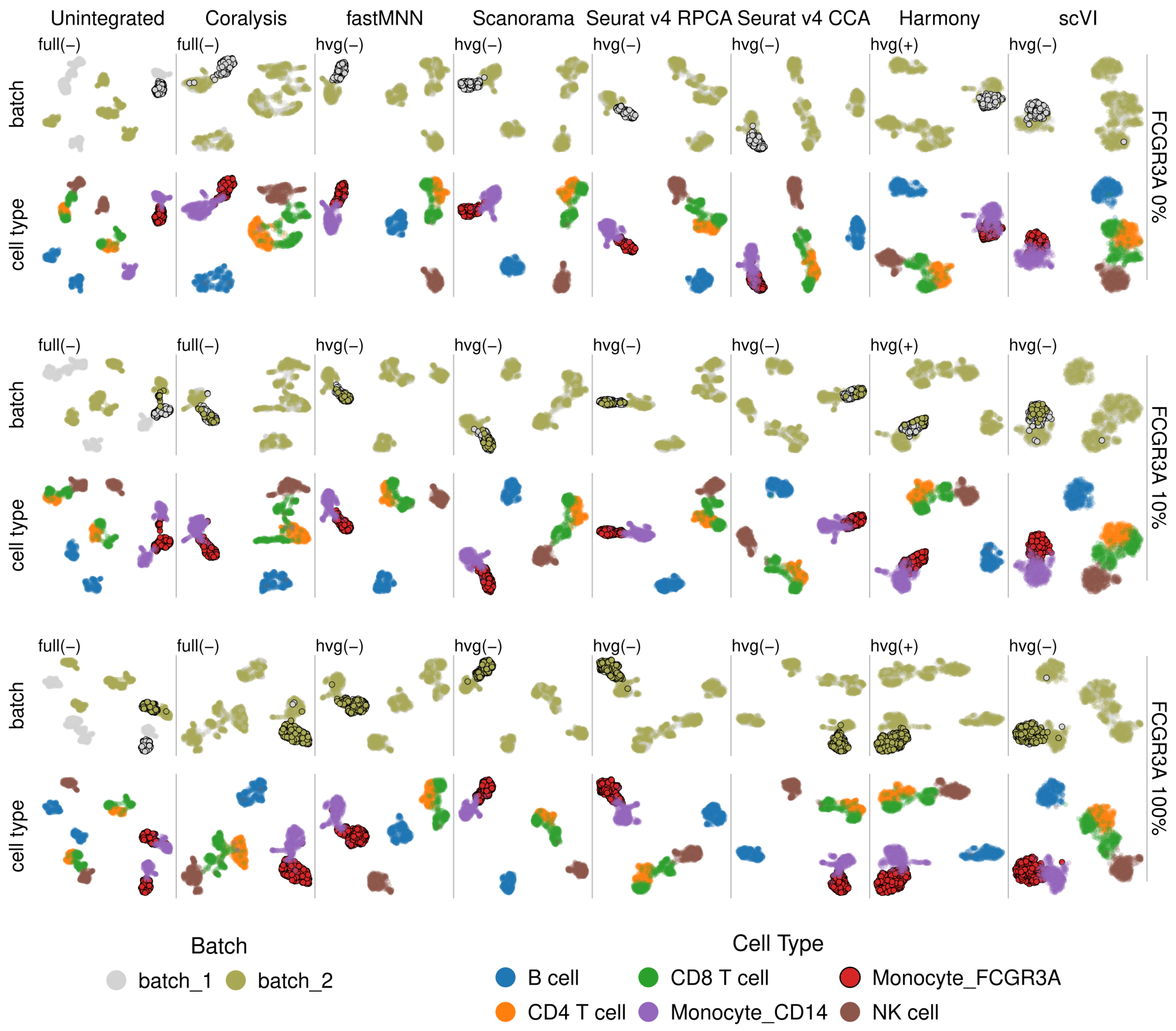

Supplementary Figure S14. UMAP projections highlighting the batch (top) and cell-type (bottom) identity for the top-performing methods, along the unintegrated projection, across the 0%, 10%, and 100% FCGR3A monocyte imbalance tasks (i.e., FCGR3A monocytes completely absent in “batch_2”, or down-sampled to 10% in “batch_2”, and fully balanced across batches).

Method	Bio conservation				Batch correction					Aggregate score		
	KMeans NMI	KMeans ARI	Silhouette label	cLISI	Silhouette batch	iLISI	KBET	Graph connectivity	PCR comparison	Batch correction	Bio conservation	Total
**Coralysis**	0.76	0.60	0.66	1.00	0.89	0.24	0.45	0.82	0.94	0.67	0.75	0.72
**Unintegrated**	0.68	0.38	0.54	1.00	0.81	0.00	0.05	0.71	0.00	0.32	0.65	0.52

Supplementary Figure S15. Assessment of integration performed with Coralysis on the ADT dataset provided by the scib-metrics python package using as ground-truth the cell-type labels given in Hao et al. [30] (at level 2 of granularity).

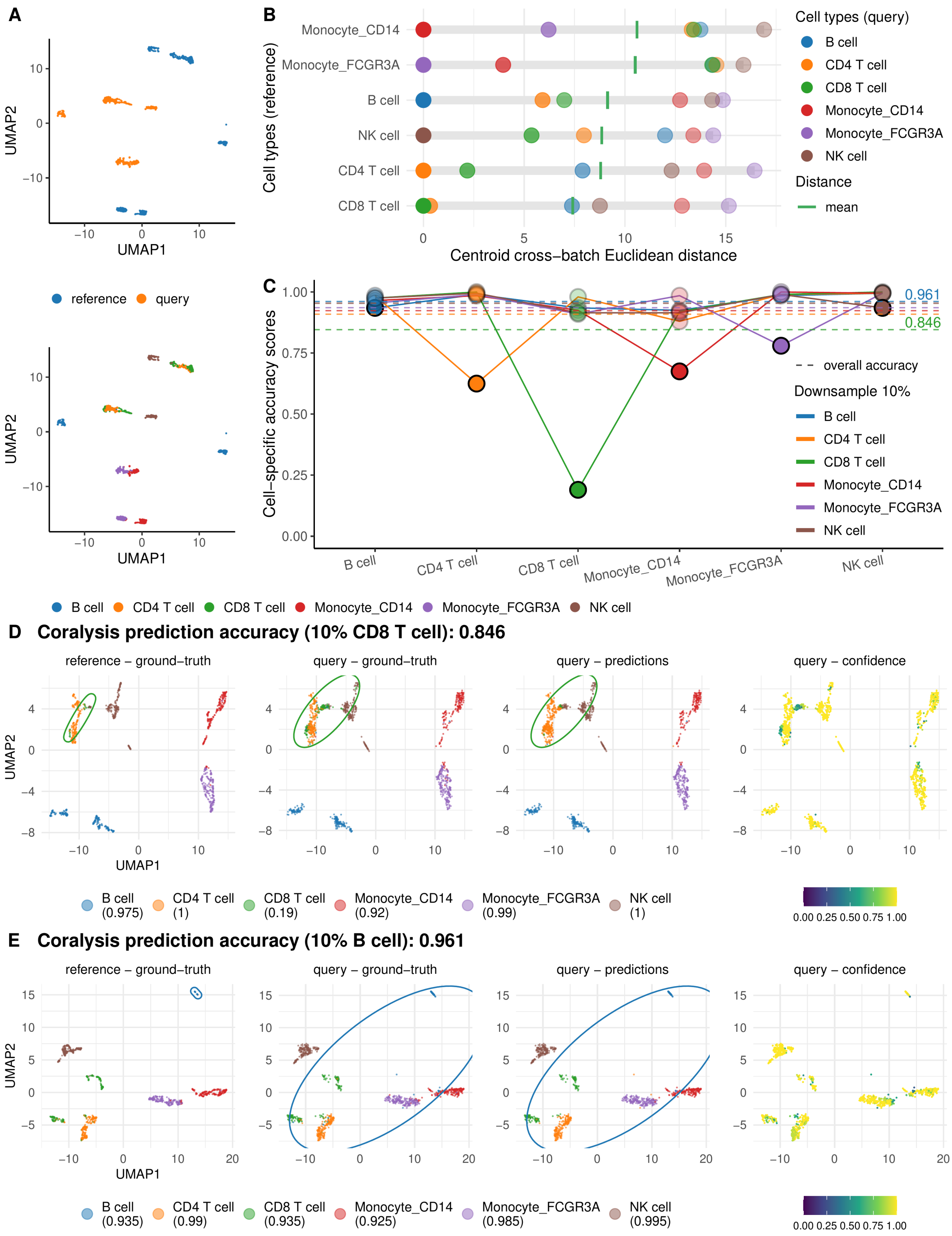
A
B
C
D
E
UMAP1
UMAP2
reference
query
Cell types (reference)
Centroid cross−batch Euclidean distance
Cell types (query)
B cell
CD4 T cell
CD8 T cell
Monocyte_CD14
Monocyte_FCGR3A
NK cell
Distance
mean
Cell−specific accuracy scores
0.961
0.846
overall accuracy
Downsample 10%
Coralysis prediction accuracy (10% CD8 T cell): 0.846
reference – ground−truth
query – ground−truth
query – predictions
query – confidence
B cell (0.975)
CD4 T cell (1)
CD8 T cell (0.19)
Monocyte_CD14 (0.92)
Monocyte_FCGR3A (0.99)
NK cell (1)
0.00 0.25 0.50 0.75 1.00
Coralysis prediction accuracy (10% B cell): 0.961
B cell (0.935)
CD4 T cell (0.99)
CD8 T cell (0.935)
Monocyte_CD14 (0.925)
Monocyte_FCGR3A (0.985)
NK cell (0.995)

Supplementary Figure S16. Accuracy performance of Coralysis reference-mapping method for a query-reference scenario of imbalanced cell types. (**A**) Unintegrated UMAP projection of query and reference PBMC datasets coloured by dataset (top) and cell-type (bottom) identities. (**B**) Centroid cross-batch Euclidean distance in PCA space highlighting the similarity between cell types across batches. The distance between the same cell type across the reference-query for every reference-query comparison was subtracted in the respective comparison in order to set the distance of every comparison to start from zero. (**C**) Cell-specific accuracy scores in down-sampled comparisons. In each reference-query comparison one cell type was down-sampled to 10%. The accuracy scores for each cell type in the same comparison are joined by solid lines, their colours representing the down-sampled cell type. The average accuracy score for each comparison is given by the dashed line, with the two values highlighted corresponding to the lowest and highest overall accuracy (CD8 T cell-B cell). For every comparison, the darkest dot corresponds to the down-sampled cell type. Projection of query onto reference UMAP highlighting ground-truth, predictions and confidence scores obtained with the Coralysis reference-mapping method for the comparisons with the lowest (**D**) and highest (**E**) overall accuracy (CD8 T cell-B cell). The confidence scores represent the proportion of K neighbours from the winning class (K=10). Ellipses circumscribe the position of the down-sampled cell type.

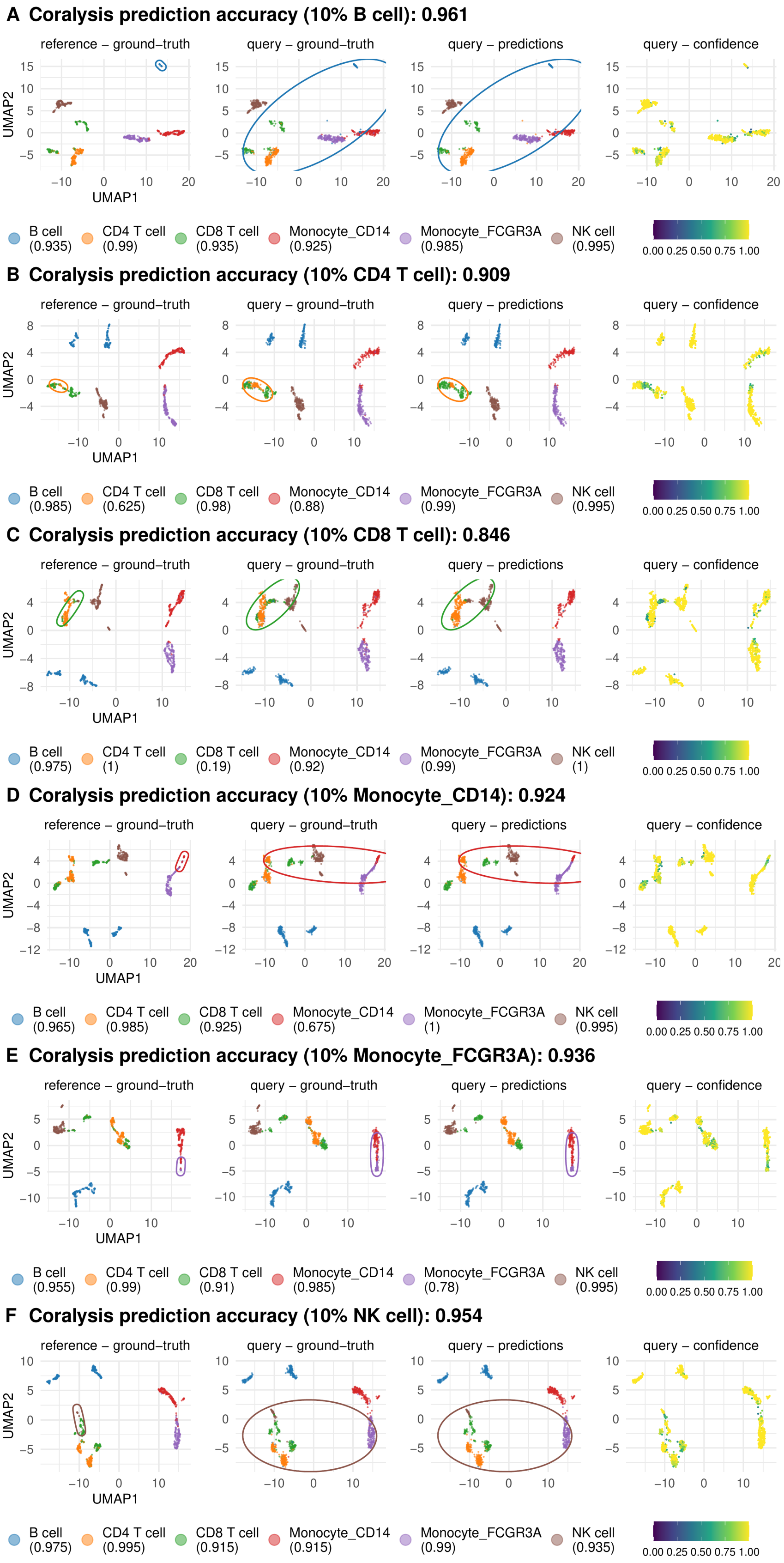
A Coralysis prediction accuracy (10% B cell): 0.961
reference – ground–truth
query – ground–truth
query – predictions
query – confidence
UMAP1
UMAP2
B cell (0.935)
CD4 T cell (0.99)
CD8 T cell (0.935)
Monocyte_CD14 (0.925)
Monocyte_FCGR3A (0.985)
NK cell (0.995)
0.00 0.25 0.50 0.75 1.00
B Coralysis prediction accuracy (10% CD4 T cell): 0.909
reference – ground–truth
query – ground–truth
query – predictions
query – confidence
UMAP1
UMAP2
B cell (0.985)
CD4 T cell (0.625)
CD8 T cell (0.98)
Monocyte_CD14 (0.88)
Monocyte_FCGR3A (0.99)
NK cell (0.995)
0.00 0.25 0.50 0.75 1.00
C Coralysis prediction accuracy (10% CD8 T cell): 0.846
reference – ground–truth
query – ground–truth
query – predictions
query – confidence
UMAP1
UMAP2
B cell (0.975)
CD4 T cell (1)
CD8 T cell (0.19)
Monocyte_CD14 (0.92)
Monocyte_FCGR3A (0.99)
NK cell (1)
0.00 0.25 0.50 0.75 1.00
D Coralysis prediction accuracy (10% Monocyte_CD14): 0.924
reference – ground–truth
query – ground–truth
query – predictions
query – confidence
UMAP1
UMAP2
B cell (0.965)
CD4 T cell (0.985)
CD8 T cell (0.925)
Monocyte_CD14 (0.675)
Monocyte_FCGR3A (1)
NK cell (0.995)
0.00 0.25 0.50 0.75 1.00
E Coralysis prediction accuracy (10% Monocyte_FCGR3A): 0.936
reference – ground–truth
query – ground–truth
query – predictions
query – confidence
UMAP1
UMAP2
B cell (0.955)
CD4 T cell (0.99)
CD8 T cell (0.91)
Monocyte_CD14 (0.985)
Monocyte_FCGR3A (0.78)
NK cell (0.995)
0.00 0.25 0.50 0.75 1.00
F Coralysis prediction accuracy (10% NK cell): 0.954
reference – ground–truth
query – ground–truth
query – predictions
query – confidence
UMAP1
UMAP2
B cell (0.975)
CD4 T cell (0.995)
CD8 T cell (0.915)
Monocyte_CD14 (0.915)
Monocyte_FCGR3A (0.99)
NK cell (0.935)
0.00 0.25 0.50 0.75 1.00

Supplementary Figure S17. Projection of query onto reference UMAP for every reference cell-type downsampled. Projection of query onto reference UMAP highlighting ground-truth, predictions and confidence scores obtained with Coralysis reference-mapping method for every reference cell-type downsampled to 10%. The confidence scores represent the proportion of K neighbors from the winning class (K=10). Ellipses circumscribe the position of the downsampled cell type.

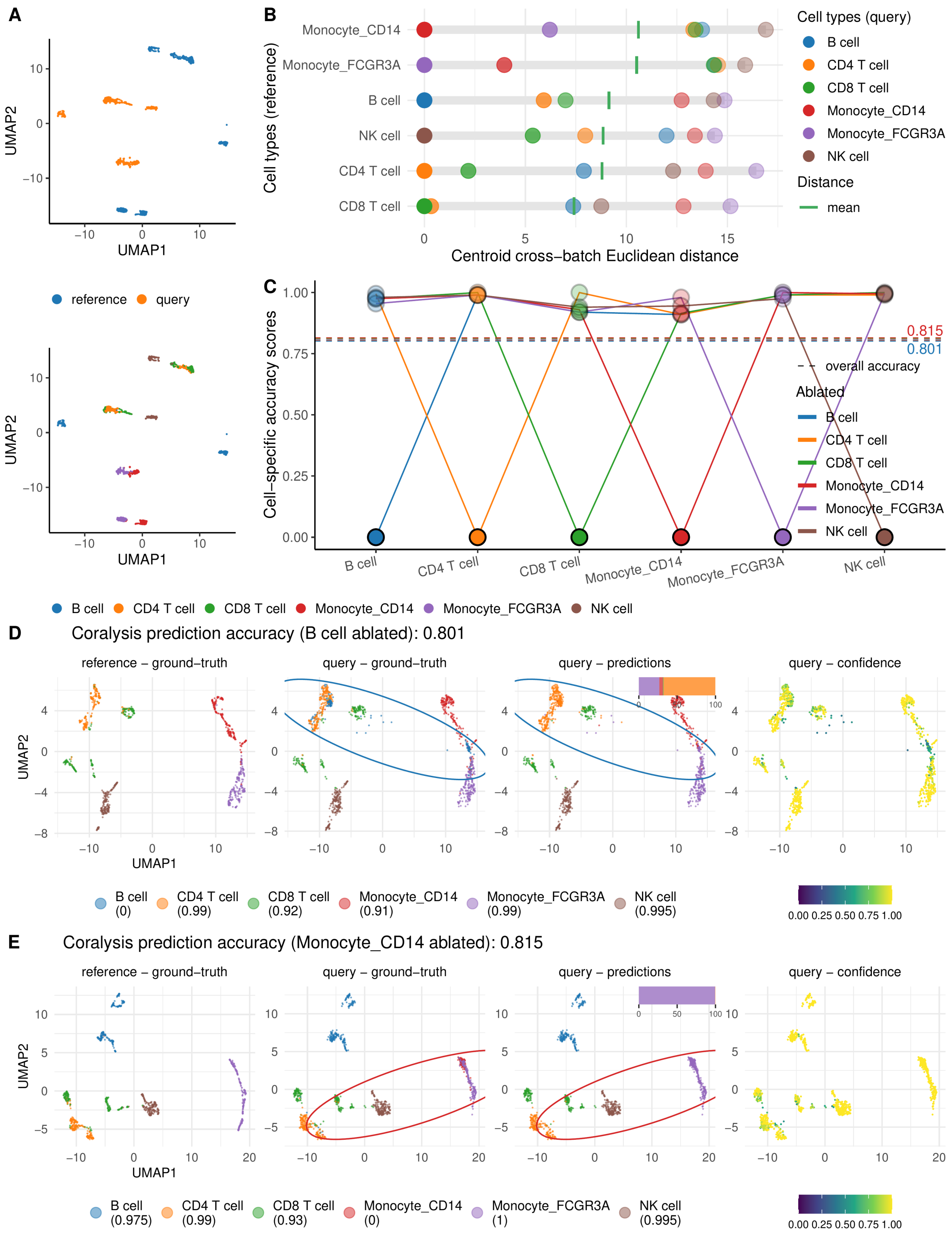
A
UMAP2
UMAP1
reference
query
B
Monocyte_CD14
Monocyte_FCGR3A
B cell
NK cell
CD4 T cell
CD8 T cell
Cell types (reference)
Centroid cross−batch Euclidean distance
Cell types (query)
Distance
mean
C
Cell−specific accuracy scores
0.815
0.801
overall accuracy
Ablated
B cell
CD4 T cell
CD8 T cell
Monocyte_CD14
Monocyte_FCGR3A
NK cell
D
Coralysis prediction accuracy (B cell ablated): 0.801
reference – ground−truth
query – ground−truth
query – predictions
query – confidence
B cell (0)
CD4 T cell (0.99)
CD8 T cell (0.92)
Monocyte_CD14 (0.91)
Monocyte_FCGR3A (0.99)
NK cell (0.995)
E
Coralysis prediction accuracy (Monocyte_CD14 ablated): 0.815
B cell (0.975)
CD4 T cell (0.99)
CD8 T cell (0.93)
Monocyte_CD14 (0)
Monocyte_FCGR3A (1)
NK cell (0.995)

Supplementary Figure S18. Accuracy performance of Coralysis reference-mapping method for a query-reference scenario of unshared cell types. (**A**) Unintegrated UMAP projection of query and reference PBMC datasets coloured by dataset (top) and cell-type (bottom) identities. (**B**) Centroid cross-batch Euclidean distance in PCA space highlighting the similarity between cell types across batches. The distance between the same cell type across the reference-query for every reference-query comparison was subtracted in the respective comparison in order to set the distance of every comparison to start from zero. (**C**) Cell-specific accuracy scores in ablated comparisons. In each reference-query comparison one cell type was ablated. The accuracy scores for each cell type in the same comparison are joined by the solid lines, their colours representing the ablated cell type. The average accuracy score for each comparison is given by the dashed line, with the two values highlighted corresponding to the lowest and highest overall accuracy (B cell–CD14 monocyte). For every comparison, the darkest dot corresponds to the ablated cell type. Projection of query onto reference UMAP highlighting ground-truth, predictions and confidence scores obtained with the Coralysis reference-mapping method for the comparisons with the lowest (**D**) and the highest (**E**) overall accuracy (B cell-CD14 monocyte). The bar plot above the UMAP corresponds to the cell type labels against which the query cell type, ablated in the reference, was classified. The confidence scores represent the proportion of K neighbours from the winning class (K=10). Ellipses circumscribe the position of the ablated cell type.

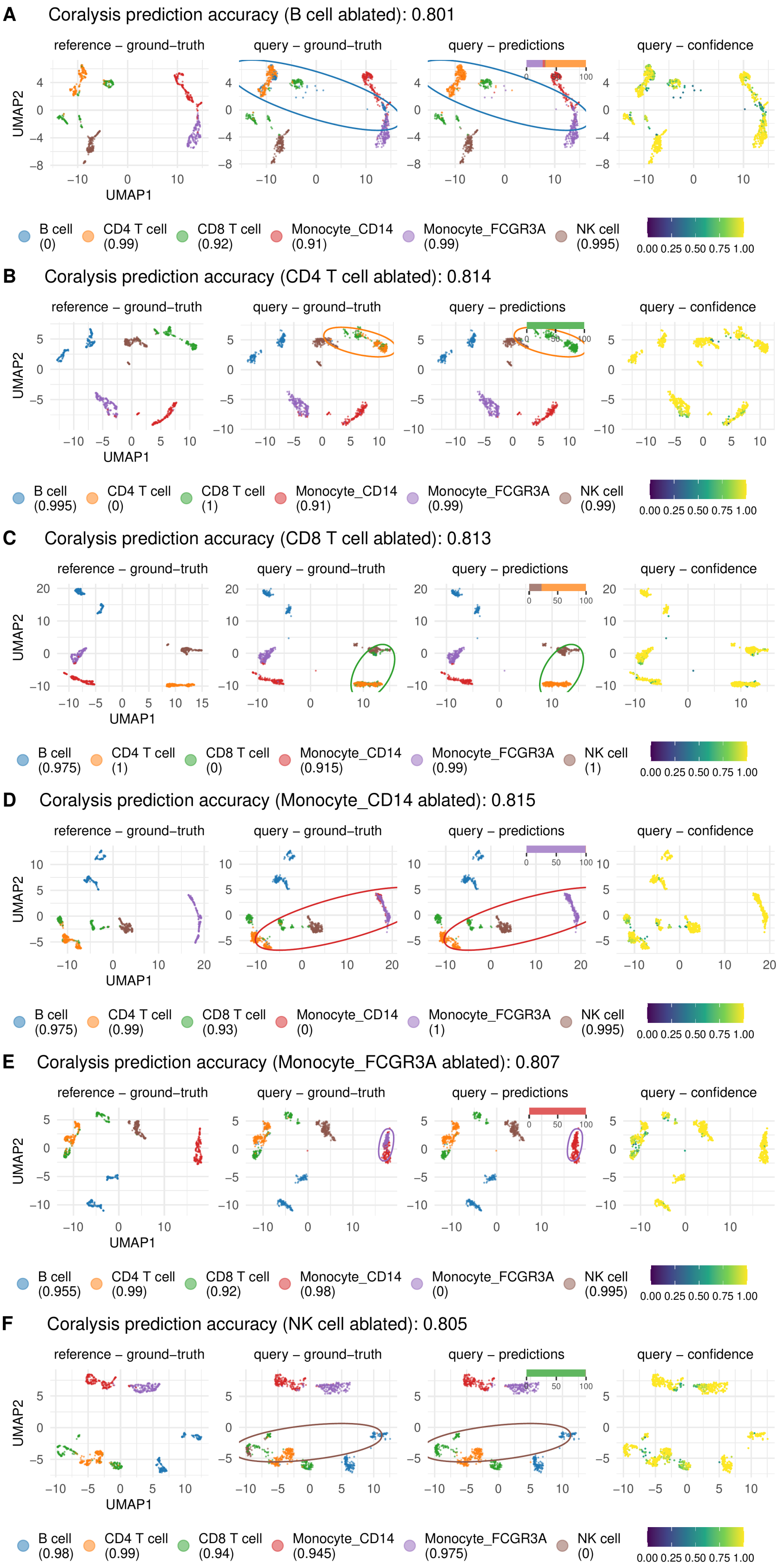
A
Coralysis prediction accuracy (B cell ablated): 0.801
reference – ground–truth
query – ground–truth
query – predictions
query – confidence
UMAP2
UMAP1
B cell (0)
CD4 T cell (0.99)
CD8 T cell (0.92)
Monocyte_CD14 (0.91)
Monocyte_FCGR3A (0.99)
NK cell (0.995)
0.00 0.25 0.50 0.75 1.00
B
Coralysis prediction accuracy (CD4 T cell ablated): 0.814
reference – ground–truth
query – ground–truth
query – predictions
query – confidence
UMAP2
UMAP1
B cell (0.995)
CD4 T cell (0)
CD8 T cell (1)
Monocyte_CD14 (0.91)
Monocyte_FCGR3A (0.99)
NK cell (0.99)
0.00 0.25 0.50 0.75 1.00
C
Coralysis prediction accuracy (CD8 T cell ablated): 0.813
reference – ground–truth
query – ground–truth
query – predictions
query – confidence
UMAP2
UMAP1
B cell (0.975)
CD4 T cell (1)
CD8 T cell (0)
Monocyte_CD14 (0.915)
Monocyte_FCGR3A (0.99)
NK cell (1)
0.00 0.25 0.50 0.75 1.00
D
Coralysis prediction accuracy (Monocyte_CD14 ablated): 0.815
reference – ground–truth
query – ground–truth
query – predictions
query – confidence
UMAP2
UMAP1
B cell (0.975)
CD4 T cell (0.99)
CD8 T cell (0.93)
Monocyte_CD14 (0)
Monocyte_FCGR3A (1)
NK cell (0.995)
0.00 0.25 0.50 0.75 1.00
E
Coralysis prediction accuracy (Monocyte_FCGR3A ablated): 0.807
reference – ground–truth
query – ground–truth
query – predictions
query – confidence
UMAP2
UMAP1
B cell (0.955)
CD4 T cell (0.99)
CD8 T cell (0.92)
Monocyte_CD14 (0.98)
Monocyte_FCGR3A (0)
NK cell (0.995)
0.00 0.25 0.50 0.75 1.00
F
Coralysis prediction accuracy (NK cell ablated): 0.805
reference – ground–truth
query – ground–truth
query – predictions
query – confidence
UMAP2
UMAP1
B cell (0.98)
CD4 T cell (0.99)
CD8 T cell (0.94)
Monocyte_CD14 (0.945)
Monocyte_FCGR3A (0.975)
NK cell (0)
0.00 0.25 0.50 0.75 1.00

Supplementary Figure S19. Projection of query onto reference UMAP for every reference cell-type ablated. Projection of query onto reference UMAP highlighting ground-truth, predictions and confidence scores obtained with Coralysis reference-mapping method for every reference cell-type downsampled to 10%. The bar plot above the UMAP corresponds to the cell-type labels against which the query cell-type, ablated in the reference, was classified. The confidence scores represent the proportion of K neighbors from the winning class (K=10). Ellipses circumscribe the position of the ablated cell type.

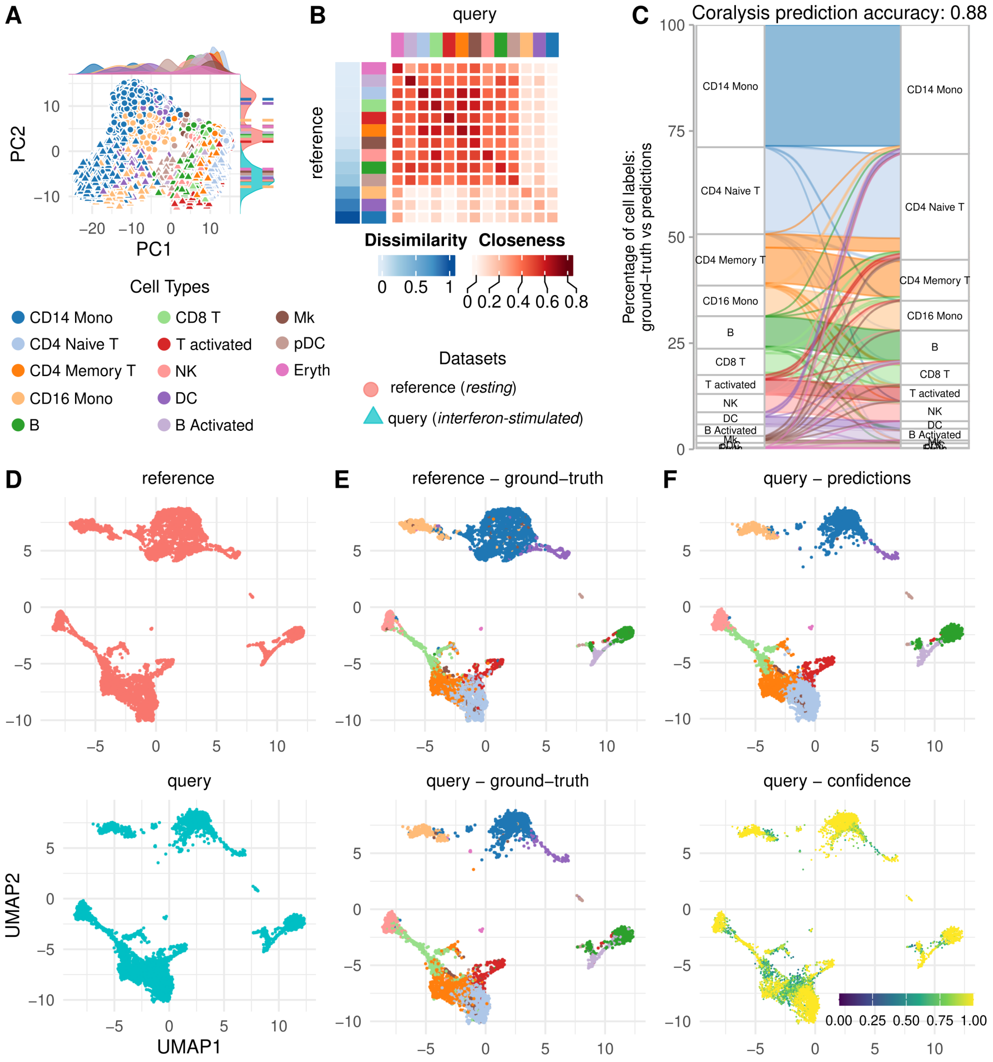

Supplementary Figure S20. Accuracy performance of Coralysis reference-mapping method for a query-reference scenario of varied strength of the batch effect. (**A**) Joint PCA of reference and query PBMC datasets. Reference and query consisted of resting and interferon-stimulated PBMCs, respectively. Shapes represent reference-query identity and colours represent cell types. Distributions at the top and left highlight cell types and dataset identity across Principal Component 1 and Principal Component 2 (PC2), respectively. Dashed lines across PC2 dataset-identity distributions correspond to the cell type centroids across reference-query. (**B**) Cross-batch centroid (Euclidean) distance between reference-query cell types. Dissimilarity corresponds to the diagonal distances min-max scaled. Closeness corresponds to the Euclidean distances scaled to fit in the range 0–1 by subtracting from 1 the distance divided by the maximum distance. (**C**) Sankey plot showing the correspondence between ground-truth cell-type labels and predictions as percentages. Query projected onto the reference UMAP highlighting the dataset identity (**D**), ground-truth cell labels (**E**) and predictions and confidence scores (**F**) between the reference and query (top–bottom). The confidence scores represent the proportion of k neighbours from the winning class (K=10).

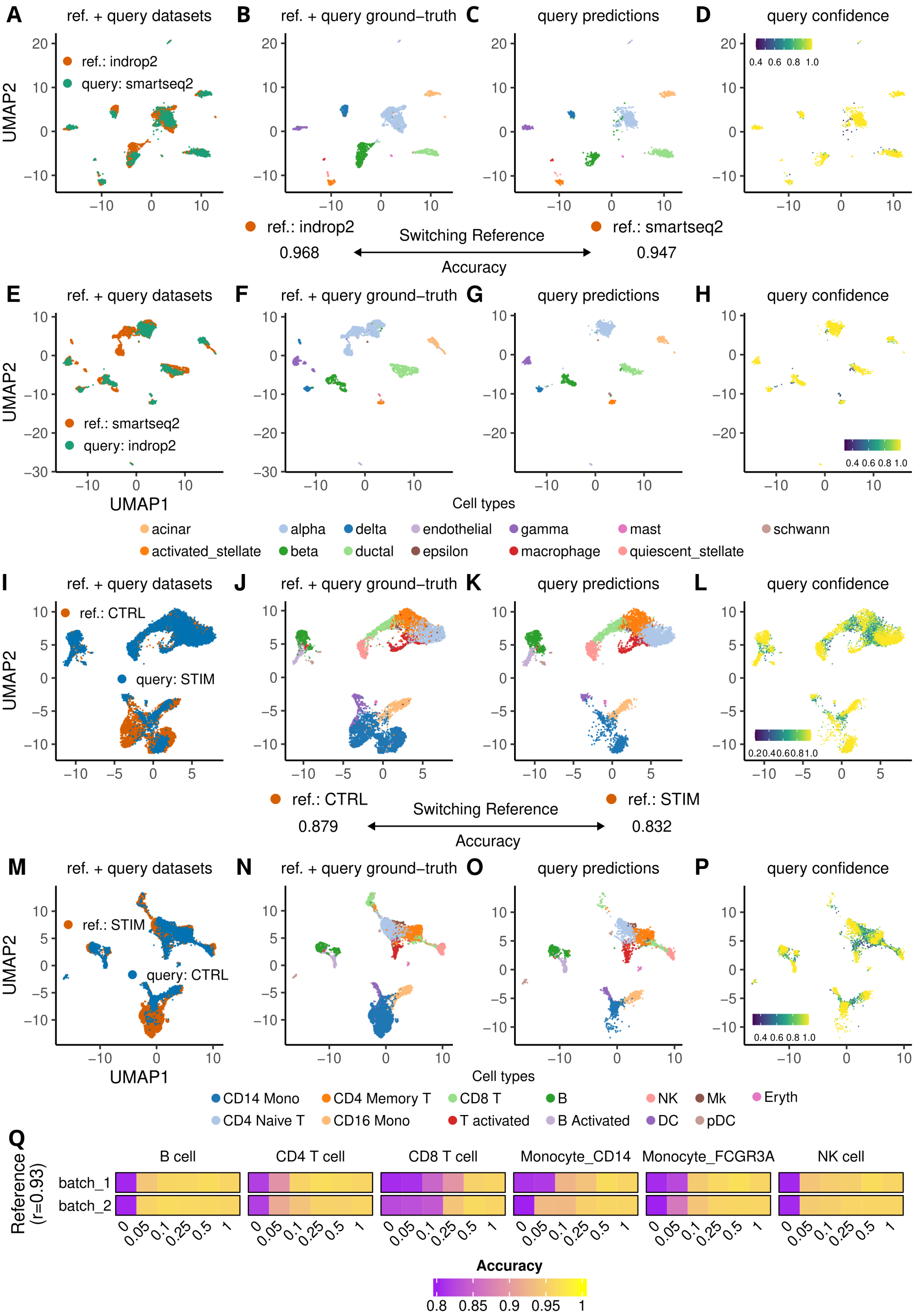

A
ref. + query datasets
B
ref. + query ground−truth
C
query predictions
D
query confidence
ref.: indrop2
query: smartseq2
UMAP2
ref.: indrop2
Switching Reference
ref.: smartseq2
0.968
0.947
Accuracy
E
F
G
H
ref.: smartseq2
query: indrop2
UMAP1
Cell types
acinar
activated_stellate
alpha
beta
delta
ductal
endothelial
epsilon
gamma
macrophage
mast
quiescent_stellate
schwann
I
J
K
L
ref.: CTRL
query: STIM
ref.: CTRL
ref.: STIM
0.879
0.832
M
N
O
P
ref.: STIM
query: CTRL
CD14 Mono
CD4 Naive T
CD4 Memory T
CD16 Mono
CD8 T
T activated
B
B Activated
NK
DC
Mk
pDC
Eryth
Q
Reference (r=0.93)
B cell
CD4 T cell
CD8 T cell
Monocyte_CD14
Monocyte_FCGR3A
NK cell
batch_1
batch_2
Accuracy

Supplementary Figure S21. Assessing the impact of switching the reference and query datasets on the accuracy of Coralysis reference-mapping. Query pancreatic cells generated using smartseq2 technology projected onto the reference UMAP of pancreatic cells generated using indrop2, highlighting reference-query identity (**A**), ground-truth cell type labels (**B**), predicted cell type labels (**C**), and confidence scores (**D**). Panels (**E–H**) show the switched scenario, with indrop2 as the query and smartseq2 as the reference. Query interferon-stimulated PBMCs projected onto the reference UMAP of resting PBMCs, highlighting reference-query identity (**I**), ground-truth cell type labels (**J**), predicted cell type labels (**K**), and confidence scores (**L**). Panels (**M–P**) show the switched scenario, with resting PBMCs as the query and interferon-stimulated PBMCs as the reference. (**Q**) Heatmap showing overall accuracy across cell type imbalance tasks, with the two PBMC batch datasets alternately used as reference or query. The top and bottom rows represent accuracy when using "batch_1" and "batch_2" as the reference, respectively. The imbalanced cell type is indicated above each heatmap. Each cell type was downsampled in the reference (left to right) to 0% (complete absence), 5%, 10%, 25%, 50%, and 100% (fully balanced).

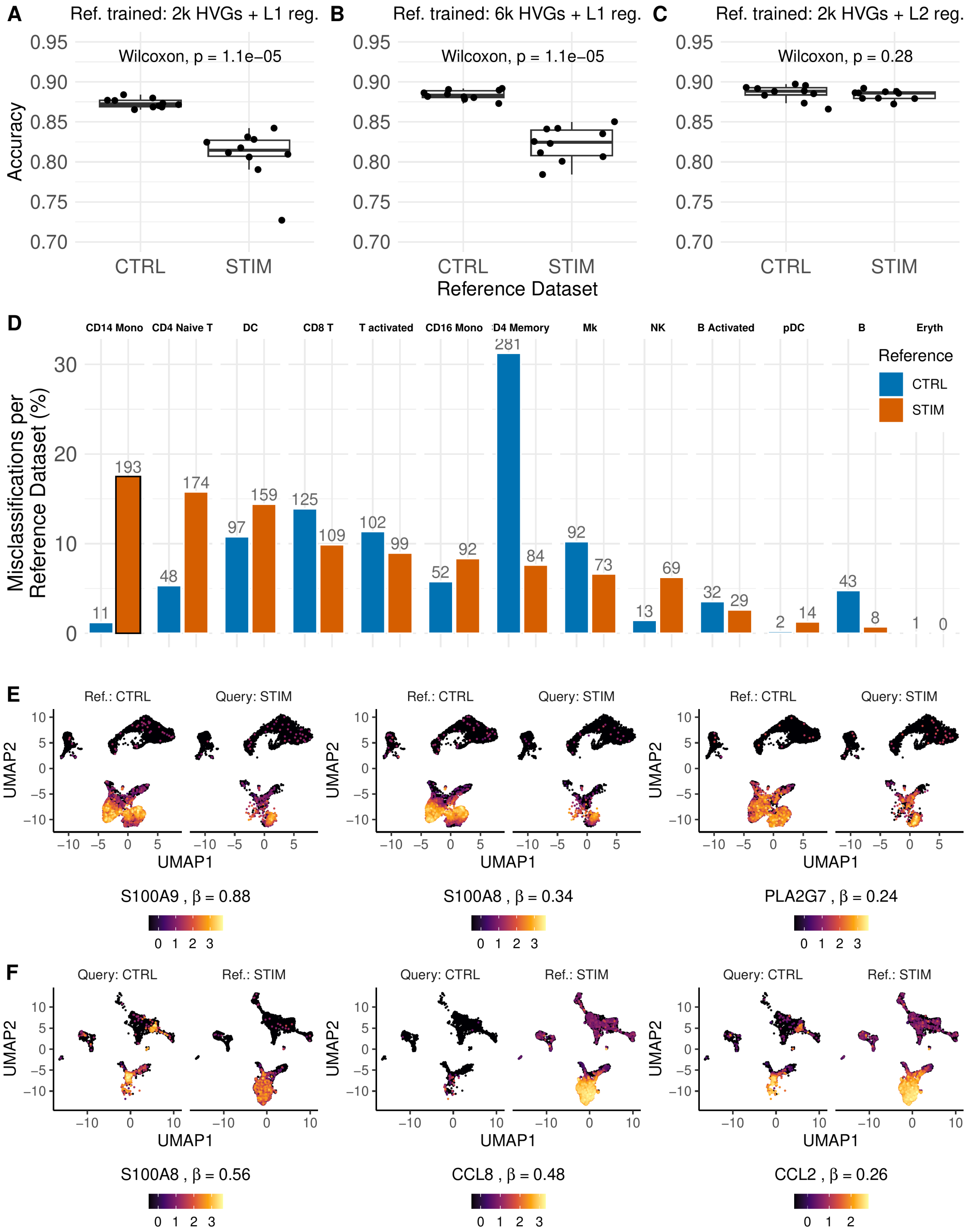
A
Ref. trained: 2k HVGs + L1 reg.
Wilcoxon, p = 1.1e−05
B
Ref. trained: 6k HVGs + L1 reg.
Wilcoxon, p = 1.1e−05
C
Ref. trained: 2k HVGs + L2 reg.
Wilcoxon, p = 0.28
Accuracy
0.95
0.90
0.85
0.80
0.75
0.70
CTRL
STIM
Reference Dataset
D
CD14 Mono
CD4 Naive T
DC
CD8 T
T activated
CD16 Mono
D4 Memory
Mk
NK
B Activated
pDC
B
Eryth
Misclassifications per Reference Dataset (%)
Reference
CTRL
STIM
11
193
48
174
97
159
125
109
102
99
52
92
281
84
92
73
13
69
32
29
2
14
43
8
1
0
E
Ref.: CTRL
Query: STIM
UMAP1
UMAP2
S100A9 , β = 0.88
S100A8 , β = 0.34
PLA2G7 , β = 0.24
F
Query: CTRL
Ref.: STIM
S100A8 , β = 0.56
CCL8 , β = 0.48
CCL2 , β = 0.26

Supplementary Figure S22. Replicability experiment with switched reference and query using resting and interferon-stimulated PBMC datasets. Variation in accuracy across ten reference-mapping experiments using either resting (CTRL) or interferon-stimulated (STIM) PBMCs as the reference, under three different settings: 2,000 highly variable genes (HVGs) and L1-regularization (**A**), 6,000 HVGs and L1-regularization (**B**), and 2,000 HVGs and L2-regularization (**C**). (**D**) Percentage of cell type misclassifications for each reference dataset. Expression levels of the top three positive coefficients highlighted on UMAPs when using resting (**E**) or interferon-stimulated (**F**) PBMCs as the reference. These coefficients correspond to the reference-mapping results shown in Supplementary Figure 21I-P.

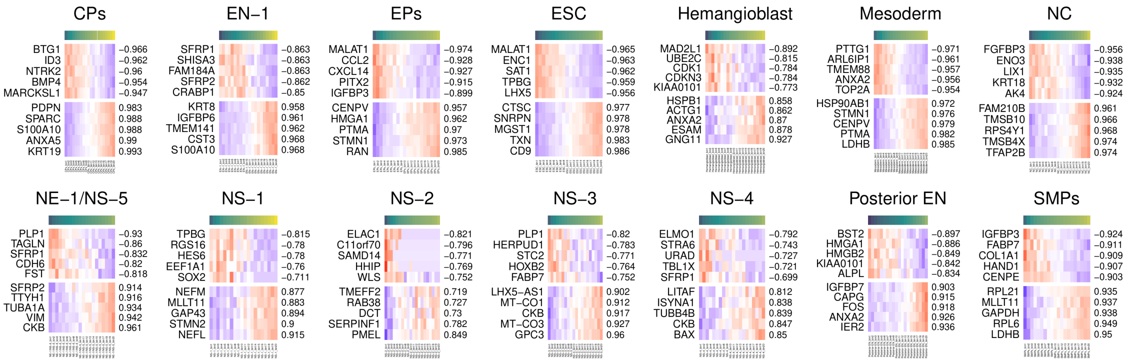

Supplementary Figure S23. Expression of the top five negative and positive correlated genes across the Coralysis cell cluster probability bins for each embryoid body cell-type. Pearson correlation was performed using the mean cell cluster probability and the average gene expression across the 20 bins of Coralysis cell cluster probability. Top color bar corresponds to the mean cell cluster probability per bin. The averaged gene expression was represented by Z-scores (by row).
